# Crystal engineering for poorly water-soluble drugs: From design to applications

**DOI:** 10.1016/j.apsb.2025.12.003

**Published:** 2025-12-05

**Authors:** An Chen, Yayun Peng, Zhuangzhuang Chen, Yi Lu, Minshan Guo, Ting Cai

**Affiliations:** State Key Laboratory of Natural Medicines, Department of Pharmaceutics, Department of Pharmaceutical Engineering, China Pharmaceutical University, Nanjing 211198, China

**Keywords:** Crystal engineering, Poorly water-soluble drugs, Aqueous solubility, Oral bioavailability, Prediction, Design, Application, AI-driven computational modeling

## Abstract

The limited aqueous solubility of active pharmaceutical ingredients (APIs) remains a major challenge in drug development, severely compromising clinical performance. Crystal engineering has emerged as a powerful and versatile approach to address this issue by rationally designing API crystal structures through precise control of intermolecular interactions, thereby enhancing solubility, dissolution rates, and ultimately bioavailability. This review systematically summarizes recent advances in crystal engineering strategies for poorly water-soluble drugs, including polymorphs, cocrystals, solvates/hydrates, nanocrystals, organic framework solids, solid solutions, liquid crystals, amorphous solids, and salts. Additionally, key challenges in translational applications are discussed, including structure-property relationship, AI-driven computational modeling, *in vitro*–*in vivo* correlation establishment, and advanced crystallization techniques. The review aims to provide strategic insights of crystal engineering for overcoming solubility barriers in next-generation drug formulations.

## Graphical abstract

This review summarizes crystal engineering techniques for enhancing solubility and bioavailability of poorly water-soluble drugs, offering strategic insights to overcome solubility barriers in next-generation formulations.

**Figure:**
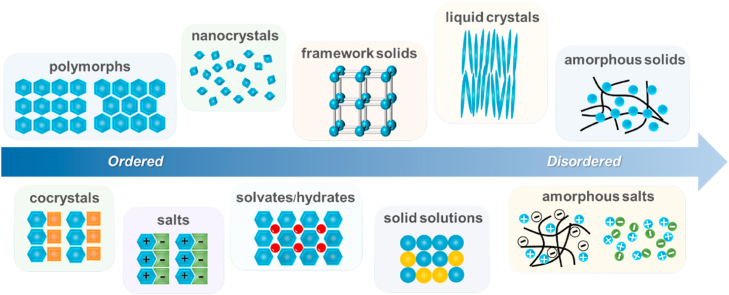

## 1 Introduction

With the advancement of high-throughput screening and combinatorial chemistry technology, coupled with a deeper understanding of receptor–ligand interactions and the intrinsic properties of drug targets, a vast number of compounds with potential medical applications have been discovered. However, most of these drug candidates are characterized by high molecular weights, lipophilicities, and low aqueous solubilities^1^. Approximately 40% of approved drugs and nearly 90% of new chemical entities in the developmental pipeline are low water solubility drugs, which are classified into biopharmaceutical classification systems (BCSs) II and IV^2^. Active pharmaceutical ingredients (APIs) with low aqueous solubility often exhibit poor absorption and limited oral bioavailability, posing significant formulation challenges that hinder drug development and compromise clinical performance^3^^,^^4^.

**Figure 1:**
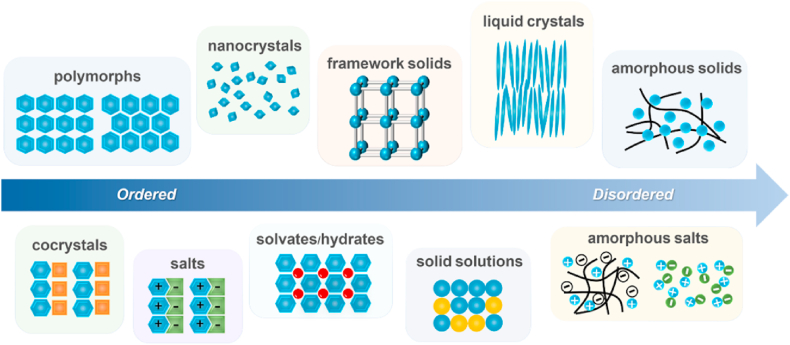
Crystal engineering approaches for poorly water-soluble drugs.

Crystal engineering was first proposed by Pepinsky in 1955, with Schmidt subsequently establishing the fundamental principles of this discipline^5^. The current phase of crystal engineering, as recognized today^6^, emerged in 1989 with Desiraju's book *Crystal Engineering: The Design of Organic Solids*, “*the understanding of intermolecular interactions in the context of crystal packing and the utilization of such understanding in the design of new solids with desired physical and chemical properties*”. For solid-state APIs, the limited aqueous solubility of pharmaceutical compounds frequently results from robust intermolecular interactions within their crystalline lattice, which hinder molecular dissociation and subsequent solvation processes^7^. Crystal engineering has emerged as an effective strategy to enhance the solubility and dissolution rate of poorly water-soluble drugs by optimizing their crystal structures while preserving their molecular structures^3^^,^^6^^,^^8^. Various crystal engineering approaches have been utilized for enhancing the solubility of drugs, including polymorphs^9^, cocrystals^10^, solvates/hydrates^11^, nanocrystals^12^, organic framework solids^13^, solid solutions^14^, liquid crystals^15^, amorphous solids^16^, and salts^17^ (Fig. 1).

This review explores recent advances in crystal engineering for enhancing the delivery of poorly soluble drugs. It covers fundamental design principles rooted in supramolecular chemistry, alongside advanced methodologies for screening, predicting physical stability, optimizing formulation design and manufacturing processes. Additionally, the review offers a critical perspective on future directions, emphasizing both emerging innovations (such as AI-driven crystal design and continuous manufacturing) and persistent challenges. These include elucidating structure–property relationships, refining preparation processes and quality control for solid forms, and enhancing the predictability of *in vitro*–*in vivo* correlations.

## 2 Polymorphs

Polymorphism is a well-established term used to describe materials with differences in crystal structure^18^. The U.S. Food and Drug Administration (FDA) defines polymorphs broadly as *different solid crystalline forms of the same substance*, under which both solvates and amorphous form are considered polymorphs^19^. In this review, the term “polymorph” refers to crystalline compounds that have identical chemical structures and molecular compositions while explicitly excluding the enantiomeric and tautomeric forms from this classification^20^. Different drug polymorphs usually exhibit distinct physicochemical properties (*e.g.*, melting point, density, solubility, and stability) due to variations in molecular stacking or conformations. These differences can further affect the manufacturing processing, bioavailability and clinical efficacy of the drug^21^.

**Figure 2:**
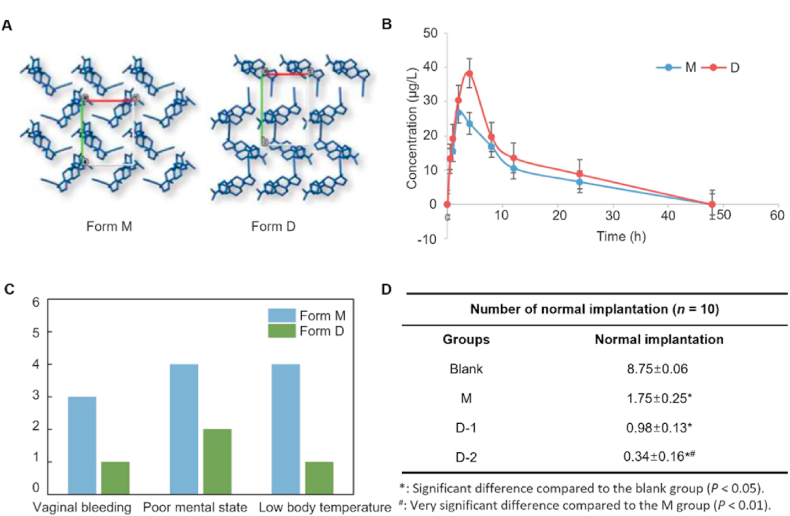
The properties of mifepristone form M and form D. (A) Crystal structures of mifepristone polymorphs. (B) Plasma mifepristone concentration–time curves (means ± SD, *n* = 10) following oral administration of mifepristone Form M and Form D to rats. (C) Adverse reactions of mifepristone polymorphs. (D) Emergency contraception pharmacodynamics of mifepristone polymorphs. Reprinted with permission from Ref. 28 Copyright © 2020, Elsevier. Reprinted with permission from Ref. 29 Copyright © 2025, Elsevier.

Polymorphs can be categorized into stable polymorphs and metastable polymorphs on the basis of differences in thermodynamic stability^22^. The metastable polymorph has a relatively high Gibbs free energy with respect to the stable form and typically has enhanced solubility, thereby providing a way for addressing the solubility challenges of poorly soluble drugs^9^^,^^23^^,^^24^. For example, bazedoxifene acetate (BA), a selective estrogen receptor modulator for postmenopausal osteoporosis, exists in three anhydrous polymorphs (Forms A, B, and D)^25^. Metastable Form A was chosen for commercialization because it offers optimal solubility and bioavailability^25^, ^26^, ^27^. Xu et al.^28^^,^^29^ reported a metastable polymorph (Form D) of the anti-progesterone agent mifepristone (Fig. 2A), which exhibited an approximately 5-fold increase in solubility and a 1.43-fold improvement in bioavailability (Fig. 2B) compared with the thermodynamically stable polymorph (Form M). Moreover, Form D demonstrated significant pharmacodynamic advantages over the stable polymorph (Fig. 2C and D)^28^^,^^29^. Further examples of drugs marketed in metastable polymorphs are summarized in^25^, ^26^, ^27^^,^^30^, ^31^, ^32^, ^33^, ^34^, ^35^, ^36^, ^37^, ^38^, ^39^ Table 1 demonstrating the successful application of metastable polymorphs in the pharmaceutical development of poorly soluble drugs.

**Table 1:** Examples of drugs marketed in metastable polymorphs.

Chemical structure	Drug	Indication	Ref.
	Aripiprazole	Schizophrenia	30
	Barbital	Sedative and hypnotic	31
	Bazedoxifene acetate	Postmenopausal osteoporosis	25, 26, 27
	Chloramphenicol palmitate	Bacterial infections	32
	Cimetidine	Stomach ulcers	33
	Dabigatran etexilate mesylate	Blood clots and stroke	34
	Famotidine	Gastric ulcers, duodenal ulcers, Zollinger–Ellison syndrome, and gastroesophageal reflux disease	35,36
	Mebendazole	Helminthiasis	37
	Ranitidine hydrochloride	Duodenal ulcers, heartburn, acid reflux, and Zollinger−Ellison syndrome	38
	Sulfamerazine	Bacterial infections	39

Polymorph screening, though widely used for discovering metastable polymorphs, remains time-consuming and labor-intensive^40^. The growing adoption of automation in high-throughput screening has emerged as an effective approach to accelerate the discovery of potential metastable polymorphs, significantly expanding polymorph selection for drug development^39^. For example, Weatherston et al.^41^ employed high-throughput encapsulated nanodroplet crystallization (ENaCt) to investigate the polymorphism of 5-methyl-2-((2-nitrophenyl)amino)thiophene-3-carbonitrile (ROY). Through an ENaCt screen of 1536 individual crystallization experiments assisted by an STP LabTech mosquito® Xtal3 liquid handling robot, the 14th ROY polymorph was identified. This discovery highlights the power of high-throughput screening in the identification and validation of elusive metastable polymorphs^41^. Moreover, complementing experimental methods, *in silico* design—particularly crystal structure prediction (CSP)—is frequently employed and continuously updated in iterative algorithms to more accurately enumerate and rank all possible polymorphs of a molecule without any a priori experimental knowledge^42^, ^43^, ^44^. CSP methods commonly include initial structure-dependent methods (basin hopping, minimum hopping, simulated annealing, and metadynamics) and *ab initio* global optimization methods (random search, particle swarm optimization, and evolutionary algorithms)^42^^,^^45^. More recently, machine learning has been integrated into CSP workflows to accelerate and support decision and clustering results, which has provided a new impetus in the CSP field^46^^,^^47^. Notably, Zhou et al.^47^ combined a novel systematic crystal packing search algorithm with machine learning force fields in a hierarchical crystal energy ranking. Validation of 66 molecules with 137 experimentally known polymorphs demonstrated superior accuracy and efficiency, establishing a robust platform to support small-molecule drug development^47^.

**Figure 3:**
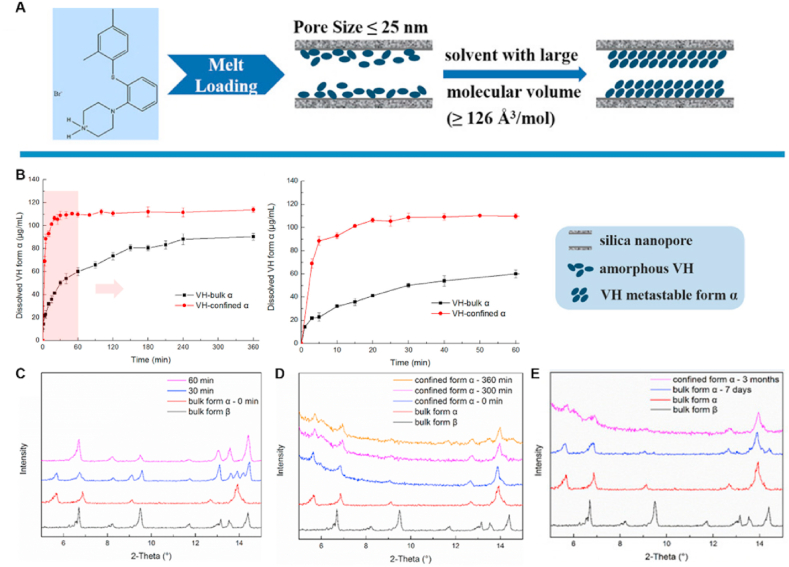
(A) Preparation of VH metastable form *α* in silica nanopores, (B) Comparative dissolution of confined VH form *α* and bulk VH form *α*. (C) PXRD patterns of suspended particles during the dissolution process of bulk VH form *α*. (D) PXRD patterns of suspended particles during the dissolution process of confined VH form *α*. (E) Physical stability of confined VH form *α* and bulk VH form *α* at 303.15 K and 75.1 ± 0.2% relative humidity. Reprinted with permission from Ref. 58 Copyright © 2022, American Chemical Society.

When metastable forms have been identified, the selective crystallization of metastable polymorphs is typically achieved using solution crystallization^48^, melt crystallization^49^, milling^50^, or sublimation^51^ methods under controlled conditions (temperature, supersaturation, solvent, seeding, additives, etc.)^52^. More recently, advanced techniques such as electric fields^53^, magnetic fields^54^, supercritical fluids^55^, surface templates^56^, and confinement^57^^,^^58^ have been developed to prepare metastable forms. Owing to their relatively high Gibbs free energy, metastable polymorphs are likely transformed into thermodynamically stable forms by solution-mediated phase transformation (SMPT) or solid–solid phase transformation (SSPT)^59^^,^^60^. Consequently, aerogels^61^, spatial confinement (nanoconfinement, coating)^58^^,^^62^, additives (organic small molecules, polymers, surfactants)^48^^,^^63^^,^^64^, and process parameters (temperature, humidity, pressure)^52^^,^^65^ have been studied to increase the physical stability of metastable polymorphs to maintain their pharmaceutical performance. Vortioxetine hydrobromide (VH), which is indicated for major depression and generalized anxiety disorders, has three anhydrous forms, of which the metastable Form *α* offers enhanced solubility but poor physical stability^58^. Cao et al.^58^ demonstrated, as shown in Fig. 3, that confining VH Form *α* within silica nanopores markedly improved its stability while preserving its solubility advantage. Owing to spatial confinement, the confined VH Form *α* reached an equilibrium solubility of 110 μg/mL within 30 min without phase transition during dissolution (Fig. 3B and D). However, bulk Form *α* equilibrated at only 80 μg/mL and converted to the more stable Form *β*, as confirmed by the powder X-ray diffraction characterization of the suspended particles (Fig. 3B and C)^58^. Additionally, the nootropic drug piracetam (PCM) Forms I and II exhibit an enantiotropic relationship with a transition temperature of 75 °C^64^. The addition of low-concentration additives, such as organic acids or polymers, can significantly decrease the solid–solid phase transition rates by up to five orders of magnitude^63^^,^^64^. This inhibition can be attributed to the interactions between the PCM and organic acids or the impediment of the polymer to PCM mobility during the polymorphic phase transforamtion^63^^,^^64^. These studies established the use of additives as a practical tool in the formulation development of metastable polymorphs, offering diverse pathways to inhibit phase transitions and achieve products with targeted physical stability.

Process analytical technology (PAT) has been applied for scientific research and industrial manufacturing for deepening process understanding, enabling real-time control, and ensuring the production of crystalline materials with precisely defined properties^66^^,^^67^. Specifically, PAT can provide information such as solute concentration, solid form, crystal size and habit during the crystallization process, facilitating the selective crystallization of the metastable polymorph^68^. Since polymorphic transformations during crystallization can yield less soluble forms and delay regulatory approval, PAT is now widely used to monitor polymorphic stability. Nicoud et al.^69^ utilized inline infrared spectroscopy and imaging probes to monitor the solute concentration and crystal habit in the crystallization process, providing a framework that helps control polymorphisms in mixed-suspension mixed-product removal (MSMPR) crystallizers, achieving the continuous preparation of metastable polymorphs of paracetamol (PRM). Similarly, Kalakech et al.^70^ utilized an inline Blaze900® probe coupled with offline FT-NIR spectroscopy to monitor the SMPT of PRM from its metastable Form II to stable Form I in isopropanol/water mixtures. By optimizing the operational parameters such as supersaturation and temperature, a longer stabilization period was achieved for the metastable Form II prior to its complete conversion to the thermodynamically stable Form I^70^.

Over the past decade, the research of polymorphic drugs has made significant advances in crystal structure prediction, selective crystallization, phase transformation and online analytical technologies, establishing a robust foundation for addressing poor drug solubility. Nevertheless, the physical stability of polymorphic drugs remains a persistent challenge: metastable polymorphs are prone to convert to their stable forms, thus undermining their solubility advantage. Future studies would benefit from integrating advanced computational modeling and artificial intelligence to identify potential polymorphs, predict their crystal structures, and optimize selective crystallization protocols. Developing transformation models that elucidate stability relationships among polymorphs and identify the key kinetic drivers will provide practical guidance for designing inhibition strategies. Finally, process analytical technology requires continuous enhancement in spectral interpretation, sensitivity and scalability to enable precise, real-time and reliable monitoring throughout the development and manufacturing process of polymorphic drugs.

## 3 Cocrystals

**Figure 4:**
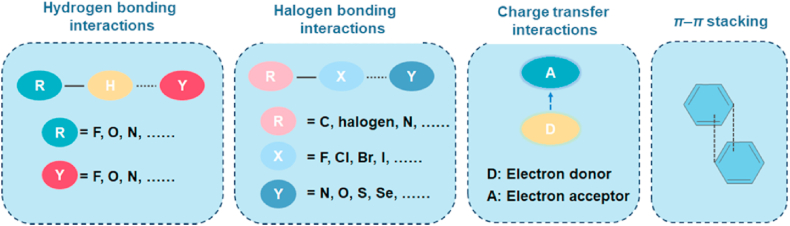
Common intermolecular interactions involved in cocrystal formation.

Over the last two decades, pharmaceutical cocrystals have become increasingly important and popular because of their potential in creating innovative multidrug formulations^71^ and enhancing the properties of pharmaceuticals^8^^,^^10^. A pharmaceutical cocrystal is generally defined as a single-crystalline phase that contains two or more different neutral molecules assembled by noncovalent interactions, such as hydrogen bonding, halogen bonding, charge transfer or *π*–*π* stacking, in a specific stoichiometric ratio, as shown in Fig. 4. At least one component is the target active pharmaceutical ingredient (API), and the coformer is either generally recognized as safe by the U.S. FDA or is another approved drug/pharmaceutical agent. Unlike salt formation, cocrystallization is applicable to any API—whether acidic, basic, or neutral—when paired with a suitable coformer^72^. This approach enhances the physicochemical properties of APIs through crystal structure modification alone, without altering the drug's chemical structure^73^. Notably, several cocrystal-based drug products, including Entresto®, Seglentis® and Mayzent®, have received regulatory approval^74^.

**Figure 5:**
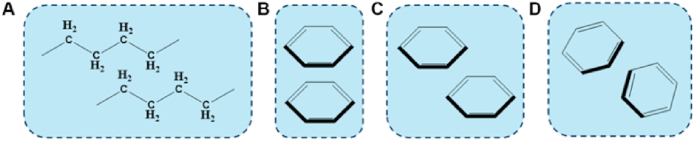
The type of packing, stacking and T-type interactions involving in stabilization of cocrystal lattices. (A) Packing of aliphatic alkane chains. (B) Eclipsed face-to-face aromatic stacking. (C) Offset face-to-face aromatic stacking. (D) Edge-to-face stacking (T-type). Reprinted with permission from Ref. 81 Copyright © 2024, The Authors.

Hydrogen bonding supramolecular synthons usually serve as a significant driving force in the formation of cocrystals because of their strength, directionality, and structural stability^75^^,^^76^. Supramolecular synthons can be categorized as homosynthons (*e.g.*, acid–acid interactions) or heterosynthons (*e.g.*, acid–amide interactions). Furthermore, halogen bonding in supramolecular synthons also plays a crucial role in cocrystal design, often occurring between halogen atoms and strongly electronegative atoms or electron-rich systems^77^, ^78^, ^79^, ^80^. Recently, Cruz-Cabeza and coworkers analyzed 3082 cocrystals (at a 1:1 M ratio) obtained from the Cambridge Structural Database and revealed that packing, stacking and T-type interactions also play key roles in the stabilization of cocrystal lattices (Fig. 5)^81^. In the analysis of the cocrystal dimer, only 20% of the interactions are strong hydrogen bonds, whereas more than half of the interactions involve stacking and T-type contacts, as shown in Fig. 5 ^81^. The packing and stacking patterns also significantly affect the cocrystal properties^82^^,^^83^. For example, the 2D-layered crystal structure of caffeine-3-nitrobenzoic acid cocrystal Form I caused plastic deformation and resulted in good tabletability^84^. Therefore, it is important to consider the interplay of both hydrogen bonding and stacking/T-type interactions when designing cocrystals rather than only focusing on supramolecular synthons.

**Figure 6:**
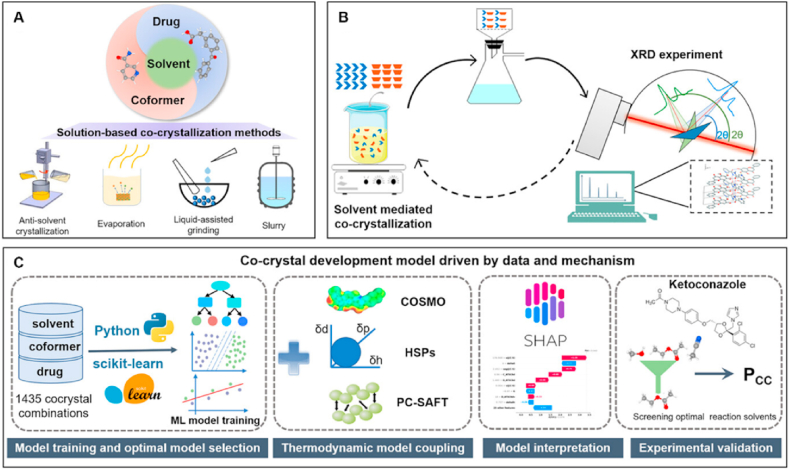
Schematic diagrams of traditional and the proposed approaches for cocrystal development. (A) Common solution-based methods for cocrystal preparation. (B) Traditional trial-and-error loop employed during the development of cocrystals. (C) Workflow employed in this study to train and analyze machine learning (ML) models to accelerate the design of cocrystals. Reprinted with permission from Ref. 100 Copyright © 2025, Wiley.

Although cocrystal engineering shows great potential for enhancing pharmaceutical performance^8^, cocrystal screening remains a major bottleneck in developing new cocrystals^85^, significantly limiting the advancement of this field. The trial-and-error method relies on extensive screening of starting materials most often used for the synthesis of cocrystals, which is time-consuming and expensive^86^. Hence, the development of a prediction method to replace the inefficient traditional approach for minimizing costs, time, and reagents is urgently needed. In the theoretical cocrystal prediction framework, knowledge-based methods and physics-based methods are usually applied. A summary of the cocrystal prediction methods is shown in Table 2 100, 101, 87, 88, 89, 90, 91, 92, 93, 94, 95, 96, 97, 98, 99. The approaches of hydrogen bond propensity (HBP)^102^^,^^103^, hydrogen bond energy (HBE) and molecular complementarity (MC)^86^^,^^104^ are knowledge-based methods. The conductor-like screening model for real solvents (COSMO-RS)^105^^,^^106^ and the Hansen solubility parameter (HSP) are physics-based methods^96^. Typically, the combination of these approaches is used for the prediction of cocrystals to improve the virtual screening efficiency and accuracy^107^. In recent years, data-driven machine learning has gained significant interest in the development of cocrystal screening models because of its advantages in terms of the automatic enhancement of optimization techniques and the ability to provide insightful recommendations from vast chemical databases^100^^,^^108^, ^109^, ^110^. Wicker et al.^111^ trained a support vector machine (SVM) algorithm model for guiding coformer selection. A model based on a combination of machine learning and COSMO-RS was established for rapid coformer screening^112^. Remarkably, the Pu group developed a graph neural network (GNN)-based deep learning model with superior performance (accuracy higher than 96%)^100^. Additionally, Song et al.^113^ combined thermodynamic modeling with machine learning to significantly improve cocrystal prediction accuracy, achieving over 90% accuracy with a coformer and solvent screening through a novel database and model integration, as shown in Fig. 6. This work demonstrated how merging mechanistic understanding with data-driven methods can advance rational cocrystal design while providing interpretable insights through SHapley Additive exPlanations (SHAP) analysis, as validated by successful ketoconazole case studies^113^. Birolo et al.^114^ developed DeepCocrystal, a deep learning framework that predicts cocrystal formation by decoding “chemical language” from a supramolecular perspective. By constructing a balanced dataset, they mitigated potential class imbalance bias and also estimated the uncertainty of model's predictions^114^. While these studies underscore machine learning's ability to provide high accuracy and rational insights for the cocrystal development, more sophisticated models are required to bridge the gap between data-driven predictions and the fundamental principles underlying cocrystal screening.

**Table 2:** Summary of cocrystal prediction methods.

Prediction method	Description	Sample	Ref.
Hydrogen bond propensity	A CCDC-developed thermodynamics-based method predicts cocrystal formation propensity (ΔPropensity ≥0 = YES; <0 = NO) by assessing API/coformer miscibility (‘like dissolves like’), balancing homomeric/heteromeric interactions *via* a logistic regression model (ROC-AUC > 0.80)	Nitrofurantoin multicomponent crystals, nevirapine– benzoic acid, cocrystal, nevirapine-3–hydroxybenzoic acid cocrystal, nevirapine– gentisic acid cocrystal, carbamazepine cocrystal	87, 88, 89
Hydrogen bond energy	Hydrogen bond energies (Δ*E* = HBE_API-coformer_−HBE_API−API/coformer-coformer_) are calculated *via* molecular electrostatic potentials (MEPs) and Hunter's parameters, where MEP maxima (*α*, hydrogen-bond donors) and minima (*β*, hydrogen-bond acceptors) determine interaction strength; -Δ*E* ≥ 11 predicts cocrystal formation (‘YES’), while -Δ*E* < 11 indicates no formation (‘NO’)	Olanzapine cocrystal and rufinamide cocrystal, CL-20 cocrystals, apigenin cocrystals	90, 91, 92
Conductor-like screening model for real solvent	This quantum chemistry-based tool predicts cocrystal formation by calculating excess enthalpy $\left(H_{\text{ex}}=H_{\text{AB}}-x_{m}H_{\text{pure}\;A}{-x}_{n}H_{\text{pure}\;B}\right)$ from molecular interactions (H-bond/van der Waals/electrostatics), where increasingly negative $H_{\text{ex}}$ values indicate higher thermodynamic stability of cocrystal	Caffeine–pyrogallol cocrystal and caffeine–phosphoric acid, piracetam cocrystal, pyrazine carboxamide cocrystal, acetazolamide cocrystal, furosemide cocrystal and nalidixic acid cocrystal	93,94
Hansen solubility parameters (HSP)	The miscibility-driven cocrystallization prediction assesses API/coformer compatibility *via* total solubility parameters ($\delta_{t}={\left(\delta_{d}^{2}+\delta_{p}^{2}+\delta_{h}^{2}\right)}^{2}$), where the solubility difference between the API and coformer is below 7 MPa^0.5^, indicates high cocrystal probability	Indomethacin cocrystal, carbamazepine cocrystal, caffeine cocrystal and theophylline cocrystal	95,96
Molecular complementarity	Cocrystal compatibility is assessed by comparing five molecular descriptors (size, shape, O/N fraction, dipole moment) between API and coformer; deviations within defined thresholds indicate high formation probability	Dipyridamole cocrystal, nevirapine cocrystal, posaconazole cocrystal	88,97,98
Machine learning	Machine learning models (*e*.*g*., SVM/RF for descriptors/fingerprints; GCNN for graphs) predict cocrystal formation by training on known cocrystal/non-cocrystal datasets, outputting probability scores (*P*) where *P* ≥ threshold yields 'YES' and *P* < threshold 'NO'	Nefiracetam cocrystal, CL-20/1-methyl-4-nitropyrazole cocrystal, and ketoprofen–carbamazepine cocrystal	100, 101, 99

Furthermore, the precise control of the cocrystallization process to achieve high product purity and yield remains a challenge in cocrystal development. Recent work by Asgarpour Khansary et al.^115^ has demonstrated the application of a molecularly enhanced computational framework for optimizing continuous pharmaceutical cocrystallization processes. They developed a novel proof-of-concept methodology that leverages density functional theory (DFT) data to extract characteristic molecular interaction fingerprints from computed Raman spectral intensities^115^. Through systematic integration of critical process parameters, including temperature, shear rate, and residence time, into a unified dimensionless *M* parameter and correlation with material composition *via* Raman intensity measurements, the team successfully established a predictive process design space^115^. This approach enables the identification of optimal operating parameters that maximize the target cocrystal yield while effectively suppressing undesirable byproducts.

Cocrystallization strategy has been widely used to improve the solubility, dissolution performance and thus oral bioavailability of poorly soluble drugs^116^, ^117^, ^118^. From a thermodynamic perspective, the incorporation of water-soluble coformers into the crystal lattice reduces the hydration barrier of hydrophobic drug cocrystals, with an extent proportional to that of the pure coformer^119^. Consequently, cocrystal solubility demonstrates a direct dependence on the coformer's solubility^119^. During the dissolution, the highly soluble coformer rapidly leaches out from the cocrystal lattice, promoting drug release and generating supersaturation of the API^120^. For example, the dissolution efficiency of quercetin–isoniazid cocrystals was 52-fold greater than that of quercetin, and the bioavailability of cocrystals was improved by 29-fold^121^.

**Figure 7:**
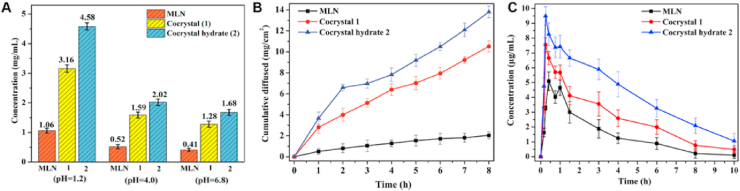
Improving the *in vitro* and *in vivo* properties of milrinone by cocrystallization with phenolic acid. (A) Solubility comparisons of milrinone, milrinone–syringic acid cocrystals (cocrystal 1) and milrinone–gallic acid hydrate cocrystals (cocrystal hydrate 2) in pH buffers 1.2, 4.0, and 6.8. (B) Cumulative amount of milrinone, cocrystal 1 and cocrystal hydrate 2 *vs* time plot. (C) Pharmacokinetic profiles with *n* = 5 rats per group. Reprinted with permission from Ref. 123 Copyright © 2022, American Chemical Society.

Milrinone, a cardiovascular drug, is a BCS IV compound with low solubility and permeability, causing poor oral bioavailability and limiting its therapeutic effect^122^. As shown in Fig. 7A–C, the cocrystal of milrinone–phenolic acid significantly improved the dissolution and permeability of milrinone, increasing its bioavailability^123^. Compared with that of pure milrinone, the relative bioavailability of milrinone–syringic acid cocrystals and milrinone–gallic acid hydrate cocrystals was 2-fold and 3.7-fold greater, respectively (Fig. 7C)^123^. Furthermore, the half-life of milrinone (2.13 h) was extended to 3.26 h for the milrinone–syringic acid cocrystal and 4.03 h for the milrinone–gallic acid hydrate cocrystal^123^.

The fixed-dose combination drug products have been increasingly used to treat some complex diseases. A cocrystal containing two therapeutic components (drug–drug cocrystal) is an ideal solid form to formulate as a fixed-dose combination product, which has shown the enhancement in solubility and dissolution rate, chemical stability and synergistic effect^8^^,^^124^. Chen et al.^125^ employed hot-melt extrusion technology for the large-scale production of celecoxib–carbamazepine drug–drug cocrystals. The cocrystal substantially modified the intrinsic dissolution profiles of the parent drugs, achieving a synchronized release of two drugs that was not observed with the physical mixture. Additionally, Song et al.^126^ developed an innovative strategy for designing multi-drug solid forms by utilizing an inorganic salt as a structural bridge to co-crystallize two distinct APIs in a ‘drug-bridge-drug’ ternary ionic cocrystal system. This approach opened new avenues for constructing advanced multi-drug delivery systems.

**Figure 8:**
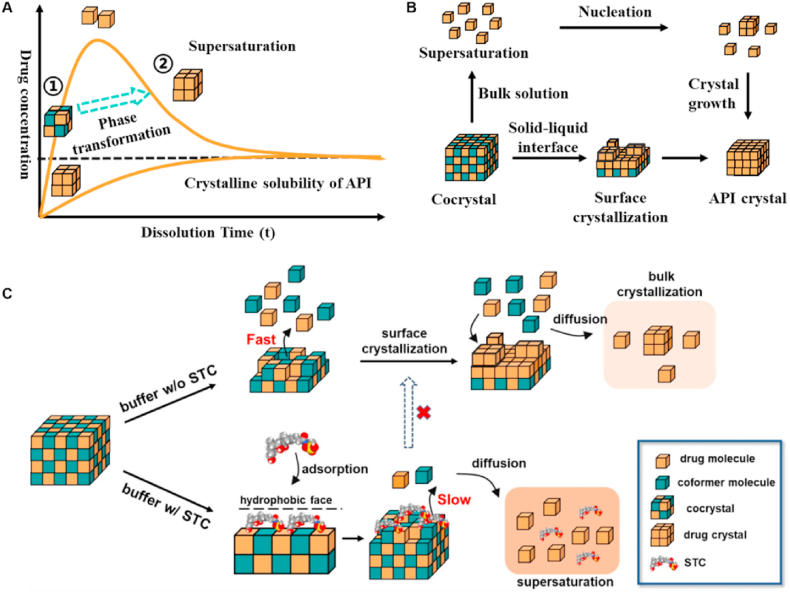
(A) Drug concentration–time profiles during cocrystal dissolution. (B) Schematic illustration of the surface and bulk phase transformation during cocrystal dissolution. (C) Schematic illustration of the effect of STC on the dissolution behaviors of IMC-SAC cocrystals. Reprinted with permission from Ref. 129 Copyright © 2022, Elsevier.

Although cocrystallization has proven highly effective in improving the oral bioavailability of poorly soluble drugs, maintaining supersaturation remains a critical challenge in cocrystal formulation development^127^. As shown in Fig. 8A, the supersaturated state generated during cocrystal dissolution is thermodynamically unstable, promoting the recrystallization of poorly soluble drugs through the SMPT^128^. The occurrence of SMPT during cocrystal dissolution proceeds *via* two pathways (Fig. 8B)^129^: (1) interfacial crystallization driven by local supersaturation at the cocrystal surface, or (2) bulk-phase crystallization occurring when the dissolved drug concentration exceeds a critical concentration. Currently, additives have been shown significant effect on preventing the phase transformation of cocrystals^130^. Rodriguez-Hornedo and co-workers revealed that the addition of surfactant solubilizer into cocrystal formulations retarded the phase transformation of danazol–vanillin cocrystals through a dual mechanism: (1) modifying the thermodynamic stability of the supersaturated state and (2) disrupting the molecular self-assembly kinetics of nucleation and crystal growth^131^. Chen et al.^129^ reported that endogenous surfactants (*e.g.*, sodium taurocholate) adsorbed on the indomethacin–saccharin cocrystal surfaces could delay coformer release. This process prevents rapid supersaturation development at the cocrystal interface, thereby reducing the crystallization driving force of the API and ultimately postponing phase transformation (Fig. 8C). Moreover, polymeric additives have been widely employed to stabilize cocrystals during dissolution by forming specific intermolecular interactions with APIs, effectively suppressing both nucleation and crystal growth^132^^,^^133^. These findings highlight the critical role of additive selection in optimizing cocrystal dissolution performance.

Despite the significant advantage of cocrystals in enhancing the solubility of poorly soluble drugs, their broader application requires addressing several key challenges. Although AI-driven prediction of coformers’ selection is now widely explored, the extensive experimental testing remains necessary to determine the physicochemical properties of resulting cocrystals. Therefore, developing robust predictive models for cocrystal properties is important to efficiently identify optimal candidates. Additionally, a deeper mechanistic understanding of cocrystal phase transformation is urgently needed to enable the rational design of stable formulations. Expanding research into the compatibility of drug–drug and drug–nutraceutical combinations is crucial to leverage their potential for bioavailability enhancement and synergistic therapeutic effects. Finally, scalable production and purification of cocrystals present a major hurdle that must be overcome to facilitate successful commercialization.

## 4 Solvates/hydrates

**Figure 9:**
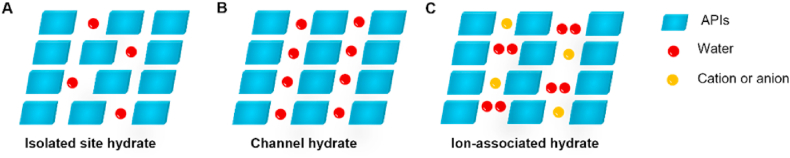
Types of hydrates: (A) Isolated site hydrate. (B) Channel hydrate. (C) Ion-associated hydrate.

Solvates are crystalline solids that incorporate solvent molecules into their crystal lattice. When the trapped solvent molecules are water molecules, these crystalline entities are termed hydrates^20^. The formation of solvates, particularly hydrates, is prevalent, in which approximately one-third of organic molecules can form hydrates^134^. The formation of hydrates or solvates is governed by specific intermolecular interactions between APIs and solvent species^135^. Water with dual hydrogen-bond donor/acceptor capabilities readily integrates into crystal lattices *via* intermolecular hydrogen bonding, making hydrates the most prevalent solvated organic compounds^135^. Based on 3258 hydrate crystal structures from the Cambridge Structural Database, Infantes et al.^136^ demonstrated that hydrate formation showed no direct correlation with hydrogen-bond donor/acceptor ratios. Instead, they identified three critical determinants in hydrate formation, including the sum/difference of total hydrogen-bond donors and acceptors, molecular polarity and presence of charged functional groups^136^^,^^137^. Solvent molecules similarly engage in strong hydrogen bonding networks with APIs or other solvents, forming dynamic clusters that stabilize the solid forms. Even weakly interacting solvent molecules can act as structural spacers (“space fillers”) within the lattice without forming strong bonds^135^. Solvate formation occurs through two primary mechanisms: (1) hydrogen-bond balance: solvent molecules compensate for mismatched H-bond donors/acceptors in API molecules^138^; (2) Packing efficiency optimization: solvent incorporation enables denser crystal packing arrangements^139^. The incorporation of solvent molecules into the crystal structure significantly alters the physicochemical properties, including the solubility, dissolution rate, and thermodynamic stability, and, in turn, the safety and efficacy of drugs^106^^,^^140^. Solvates can serve as intermediates for obtaining target polymorphs, as certain polymorphs are accessible only through the desolvation of specific solvates^141^^,^^142^. Solvates can be classified into stoichiometric and nonstoichiometric types on the basis of whether the ratio of solvent molecules to other components in the crystal lattice is fixed. Additionally, the classification of hydrates into isolated, channel and ion-coordinated hydrates (Fig. 9), which are based on the interaction types between drug and water molecules, is also widely recognized^143^.

**Figure 10:**
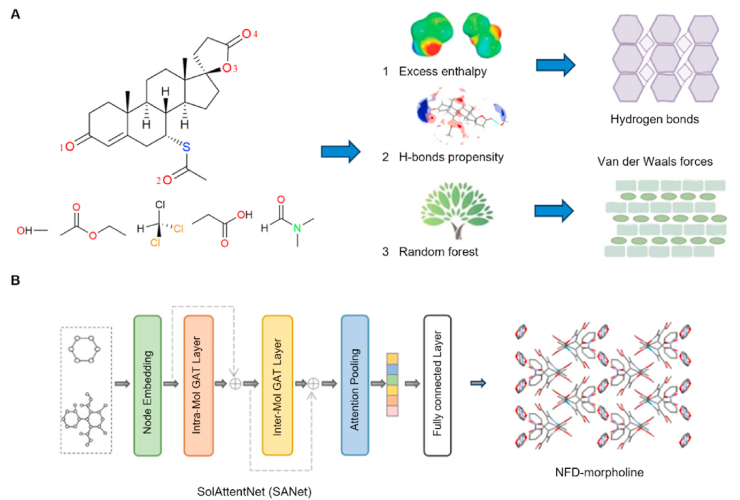
Prediction methods of solvate formation. (A) Schematic diagram illustrating the application of excess enthalpy (Hex), hydrogen bond propensity (HBP), and the random forest model (RF) for the prediction of spironolactone (SPI) in 29 solvents. Reprinted with permission from Ref. 147 Copyright © 2023, American Chemical Society. (B) Schematic framework of the attention mechanism-based GNN model for predicting the formation of nifedipine (NFD) solvates. Reprinted with permission from Ref. 148 Copyright © 2025, American Chemical Society.

Currently, a variety of methods have been employed to predict the formation probability of solvates/hydrates in different solvents, such as COSMO-RS, HBP, machine learning, MEP, and CSP. MEP helps understand the formation of hydrates or solvates by visualizing the charge distribution around a molecule, thereby predicting its interactions with water or other solvent molecules. For example, Bajpai et al.^144^ used MEP analysis to study *N*-heterocyclic aromatic compounds and found that molecules with more negative potentials at hydrogen bond acceptor sites exhibited a higher tendency for hydrate formation. CSP has also been widely used to study solvates and hydrates. Braun et al.^145^ applied CSP to analyze alkaloids such as strychnine and brucine, revealing why brucine forms multiple hydrates whereas strychnine does not. The study utilized computational tools including CrystalPredictor and CASTEP for detailed analyses. Hong et al.^146^ developed a novel CSP approach (Mapping Approach for Crystal Hydrates, MACH) that efficiently predicted hydrate structures without requiring pre-specifying water content. This method was successfully validated on compounds such as brucine dihydrate. Furthermore, Jia et al.^147^ predicted the propensity of spironolactone to form solvates in 29 solvents by the COSMO-RS, HBP, and random forest (RF) methods (Fig. 10A). Although the HBP model demonstrated an 81% accuracy in predicting solvate formation for solvents containing hydrogen bond donors, its predictive capability is significantly limited for aprotic solvent systems. Compared with the other methods, the RF method exhibited superior reliability, which was highly dependent on the quality of the training dataset. As shown in Fig. 10B, Sun et al.^148^ also developed a graph neural network model (SANet) based on attention mechanisms to predict the solvate formation of nifedipine (NFD) with 80.6% balanced accuracy. In particular, the model shows superior performance in identifying nifedipine solvates with 96% accuracy^148^.

**Figure 11:**
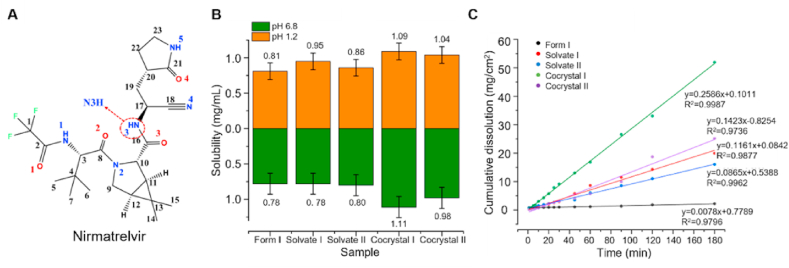
The dissolution and solubility performance of nirmatrelvir solvates. (A) Chemical structure of nirmatrelvir. (B, C) Solubility and dissolution of nirmatrelvir solvates. Reprinted with permission from Ref. 10 Copyright © 2024, Elsevier.

Some studies have shown that solvates/hydrates display greater solubility and faster dissolution performance than anhydrous forms^11^^,^^149^. Li et al.^11^ synthesized two solvates of nirmatrelvir with methyl *tert*-butyl ether (MTBE, Solvate I) and isobutyl acetate (IBAC, Solvate II) (Fig. 11A). The IDR results indicated that Form I exhibited the lowest dissolution rate (Fig. 11B and C), whereas the solvates (Solvates I and II) showed relatively high dissolution rates (Fig. 11C). Similarly, in the case of rebamipide, the formation of solvates with ethanol and dichloromethane led to marked improvements in both solubility and dissolution rate^150^. This enhancement can be attributed to the incorporation of small solvent molecules into the crystal lattice, which effectively reduces the solvation energy barrier and thus facilitates drug dissolution^150^. Meloxicam (MLX) is classified as a Class II drug, a nonsteroidal anti-inflammatory drug (NSAID), which is mainly used for the treatment of rheumatoid arthritis and osteoarthritis^151^. Five crystalline forms have been identified for MLX, with Form IV being a monohydrate^149^. The dissolution studies show that Form IV (monohydrate) exhibits superior dissolution performance, with an intrinsic dissolution rate (IDR) of 0.226 ± 0.004 mg/cm^2^/min, which is 2.3-fold greater than that of the commercially available anhydrous Form I (0.1005 ± 0.003 mg/cm^2^/min)^149^.

**Figure 12:**
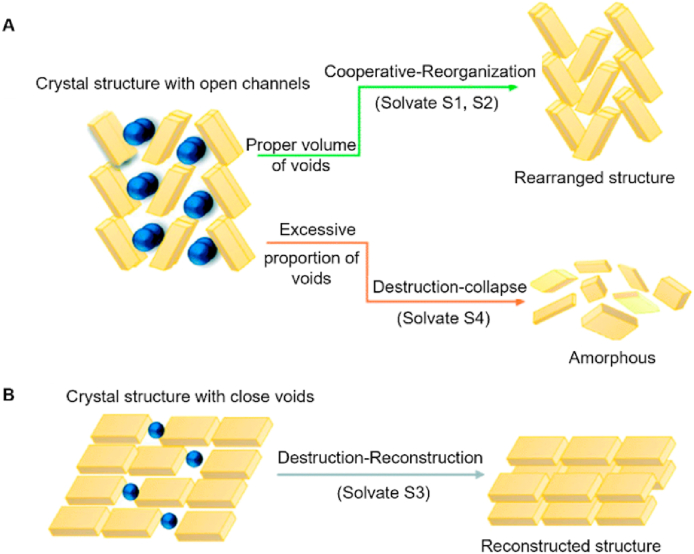
Desolvation mechanisms of cefathiamidine solvates corresponding to their crystal structures. (A) Cooperative reorganization mechanism and destruction-collapse mechanism. (B) Destruction-reconstruction. Reprinted with permission from Ref. 156 Copyright © 2021, The Royal Society of Chemistry.

When formulating a drug substance as a solvate or hydrate, it is crucial to assess the risks of desolvation or dehydration, as these processes can significantly compromise crystalline purity and therapeutic efficacy^152^^,^^153^. A notable example occurred in 2010 when 1.5 million tablets of warfarin sodium isopropyl alcohol solvate (marketed as Coumadin®) were recalled due to batch inconsistencies caused by variable isopropyl alcohol content resulting from desolvation^154^. It was found that the desolvation/dehydration typically proceeds through either cooperative or destructive pathways, depending on the nature of intermolecular interactions and the crystal lattice structure^155^. As illustrated in Fig. 12, the desolvation/dehydration behavior of crystals with open channels is determined by their void space characteristics (Fig. 12A): (1) when sufficient void space exists, solvent molecules can escape cooperatively while the crystal structure undergoes concomitant rearrangement (cooperative reorganization mechanism); (2) in cases of insufficient void space, solvent escape leads to destructive lattice collapse, ultimately yielding an amorphous solid (destruction–collapse mechanism)^156^. For crystals containing isolated closed voids, solvent removal first causes temporary structural damage before the lattice can reconstruct itself (destruction–reconstruction mechanism, Fig. 12B)^156^.

Modafinil, a drug used to treat excessive sleepiness, can form solvates with chloroform and acetonitrile^157^. Studies revealed that the desolvation mechanism of modafinil solvates is temperature-dependent, exhibiting two distinct pathways: (1) when the desolvation temperature is below the glass transition temperature (*T*_g_) of anhydrous modafinil, desolvation occurred through a nucleation–growth mechanism starting from the surface of crystals; (2) when desolvation temperature exceeds *T*_g_, desolvation proceeds *via* a cooperative nucleation pathway, resulting in the formation of a pure phase exhibiting structural filiations with the mother phase^157^. Naproxen sodium, a nonsteroidal anti-inflammatory drug commonly used in the treatment of rheumatoid arthritis, has several solid-state forms: an anhydrous form, a monohydrate, two dihydrate polymorphs, and a tetrahydrate^158^. Thakral et al.^158^ found that the presence of PVP-K12 delayed the dehydration of sodium naproxen dihydrate due to its high moisture absorption and desorption capacity, which helps maintain the hydrated state of naproxen by releasing water as needed. In contrast, microcrystalline cellulose (MCC) accelerated the dehydration process due to its fibrillar structure, which promotes capillary condensation and effectively traps sorbed water within its network when water is released from the hydrate^158^. These studies demonstrated that optimization of both processing parameters, storage conditions and excipient selection are critically important for maintaining the physical stability of solvates/hydrates during formulation development.

Douillet et al.^159^ highlighted the challenges in developing solvates of APIs, identifying solvent toxicity as a critical selection criterion. They emphasized that a solvate can only be considered viable if its residual solvent content remains below the established acceptable daily exposure limits for patient safety^159^. In a case study, Chadha et al.^160^ characterized atorvastatin calcium solvates, further confirming their compliance with residual solvent limits stipulated by the ICH Q3C Guideline. This guideline classifies solvents based on toxicity (*e.g.*, neurotoxicity, carcinogenicity) and sets permissible limits, underscoring that the toxicological risks associated with residual solvents must be rigorously assessed during solvate development. Furthermore, both hydrates and solvates present significant formulation stability and safety challenges due to their inherent risks of dehydration and desolvation. A deep understanding of the underlying mechanisms driving these processes is crucial. Additionally, the influence of excipients and manufacturing processes on dehydration/desolvation kinetics requires thorough investigation. Stability during storage is also critically impacted by environmental factors, particularly temperature and humidity (often linked to pressure). Consequently, a key future research direction is the development of robust predictive models capable of accurately forecasting the thermodynamic stability of hydrates and solvates under relevant processing and storage conditions. Such models are essential for rational formulation design and ensuring product quality throughout the lifecycle.

## 5 Nanocrystals

Pharmaceutical nanocrystals are typically defined as crystals with a particle size of less than 1 μm that can be administered *via* various routes (*e.g.*, oral, parenteral, transdermal, ocular, intranasal, and pulmonary)^161^^,^^162^. Owing to their small particle size, pharmaceutical nanocrystals exhibit increased saturation solubility, an enhanced dissolution rate, and improved adhesiveness to physiological barriers^161^^,^^163^. Moreover, pharmaceutical nanocrystals offer higher drug-loading capacity, lower manufacturing costs, and enhanced scalability compared to other nanoparticle-based carriers. These distinct advantages make nanocrystals widely applicable in overcoming the challenges associated with formulation development and the clinical translation of poorly water-soluble drugs.

**Figure 13:**
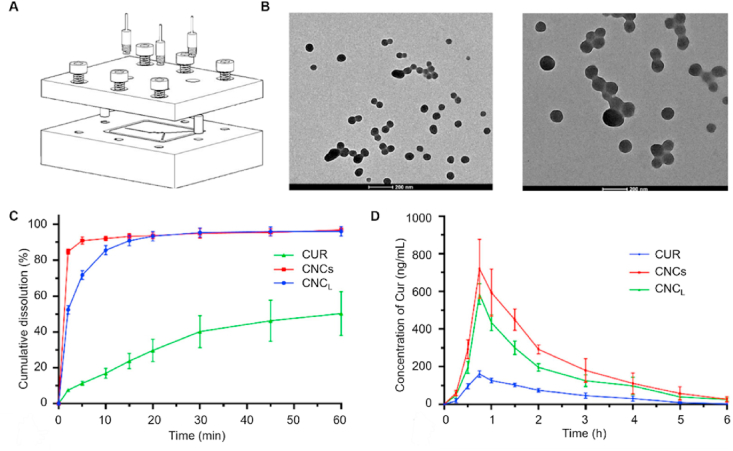
Preparation and evaluation of curcumin nanocrystals. (A) The appearance design of the stainless-steel microfluidic chip. (B) TEM images of CNC_S_ (left) and CNC_L_ (right). (C) Dissolution profiles of the curcumin from the CNC_S_ and CNC_L_ in 0.1 mol/L HCl. (D) Plasma concentration–time curves of the curcumin powder, CNC_S_, and CNC_L_ in Sprague Dawley rats after oral administration. Reprinted with permission from Ref. 174 Copyright © 2024, Elsevier.

Pharmaceutical nanocrystals are commonly prepared *via* top-down and bottom-up technologies^164^^,^^165^. Top-down methods breakdown drug particles to the nanometer size range *via* friction, whereas bottom-up techniques involve the crystallization of nanocrystals from liquids^166^. High-pressure homogenization and wet milling have become the most widely used methods for large-scale production because of their simplicity, robustness and scalability^166^. Nevertheless, these approaches suffer from issues such as residual milling media, heterogeneous particle-size distributions, and compromised stability arising from high-energy processing. Ongoing efforts focus on developing robust and scalable protocols that reliably produce nanocrystals with narrow size dispersity, enhanced physicochemical stability and acceptable safety. In recent years, microfluidization^167^, extrusion^168^, 3D printing^169^, SmartCrystals technology^170^, supercritical fluid^171^ and metastable zone-based techniques^172^, have been explored to achieve superior modulation over nanocrystal product properties. For example, Zhao et al.^173^ employed hot-melt extrusion to fabricate curcumin (CUR) nanocrystals with particle sizes ranging from 50 to 150 nm. These nanocrystals achieved rapid drug release of 80% within 2 min and resulted in a 4.07-fold increase in the AUC_0–∞_ of the metabolites compared with that in the CUR group^173^. Similarly, Zheng et al.^174^ utilized microfluidization technology (Fig. 13A) to prepare curcumin nanocrystals with particle sizes of 59.29 nm (CNC_S_) and 168.40 nm (CNC_L_) (Fig. 13B), which resulted in a rapid dissolution (Figs. 13C) and 4.26- and 3.14-fold increases in the AUC_0–∞_ (Fig. 13D), respectively, compared with those of bulk curcumin powder. These results demonstrate the excellent robustness and controllability afforded by microfluidization, underscoring its potential for future commercial fabrication of drug nanocrystals.

**Figure 14:**
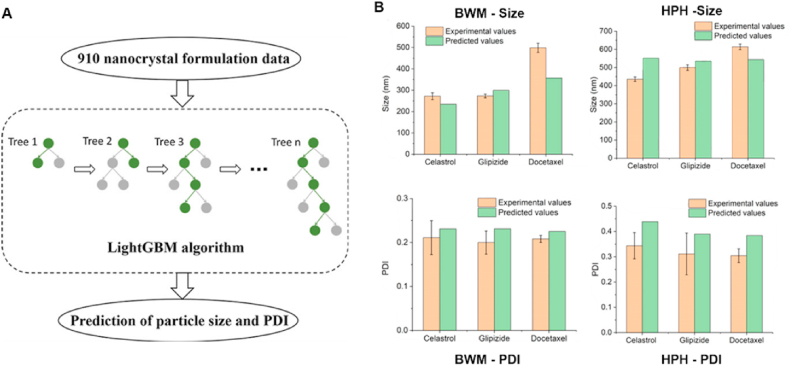
(A) Workflow of LightGBM algorithm for forecasting particle size and PDI of nanocrystals. (B) Comparisons of predicted values calculated by LightGBM with experimental size and PDI for nanocrystals prepared by BWM (left) and HPH (right). Reprinted with permission from Ref. 176 Copyright © 2020, Elsevier.

The formulation design and processing optimization of pharmaceutical nanocrystals are critical, as nanocrystals are prone to aggregation and Ostwald ripening. Traditionally, nanocrystal development has relied on empirical, trial-and-error approaches that are inefficient and lack predictive capability. Recent advances have incorporated mathematical models, computational simulations, and machine learning to facilitate nanocrystal development^175^, ^176^, ^177^, ^178^. For example, the light gradient boosting machine (lightGBM), a machine learning algorithm known for its high computational efficiency and accuracy^179^, was utilized by He et al.^176^ to identify the critical factors in nanocrystal preparation *via* ball wet milling (BWM) and high-pressure homogenization (HPH) (Fig. 14A). The lightGBM exhibits superior generalizability and accuracy in predicting nanocrystal particle size and polydispersity index (PDI), as validated using three model drugs: celastrol, glipizide and docetaxel (Fig. 14B). Recently, the same group also developed a Formulation DT platform as the pivotal module for *in silico* formulation design^180^. Latham et al.^181^ conducted molecular dynamics simulations to elucidate the role of hydrophobic interactions in driving excipient adsorption onto drug surfaces, and proposed that the fraction of the polar surface area could serve as a predictor of nanosuspension stability. These studies provide valuable strategies for rational nanocrystal formulation design and process optimization.

Pharmaceutical nanocrystal technology has been validated as an effective strategy for addressing the critical biopharmaceutical challenges associated with poorly water-soluble drugs. Owing to their nanoscale dimensions, nanocrystals markedly increase drug solubility and dissolution performance, thereby improving oral bioavailability^12^^,^^182^. Etoposide (ETO) is an antitumor drug approved for treating testicular and small cell lung cancer. However, ETO has limited oral absorption due to its poor water solubility and permeability^183^. Wang and coworkers^12^ developed a nanocrystal-loaded lipid carrier, ETO (ETO-EU@Lipo@NCs), to improve its oral absorption and anticancer efficacy. ETO-nanocrystals and ETO-EU@Lipo@NCs both exhibited higher AUC_0–*t*_ values, 5.6 and 11.0 times higher than those of ETC coarse crystals, demonstrating that particle size reduction plays an important role in improving bioavailability^12^. Furthermore, compared with the incorporation of ETO nanocrystals, the incorporation of lipid-based strategies with nanocrystals results in enhanced cellular uptake, transport, and inhibitory effects on tumors^12^.

**Figure 15:**
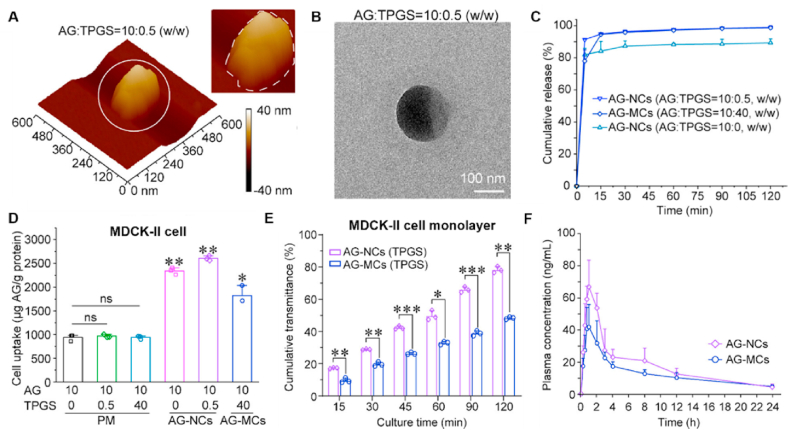
Nanocrystals of poorly water-soluble andrographolide for enhanced drug delivery. (A, B) Morphology of the AG-NCs observed *via* AFM and TEM. (C) *In vitro* dissolution of AG-NCs and AG-MCs. (D, E) Cellular uptake and transmembrane transport of AG-NCs in MDCK-II cells. (F) Plasma concentration‒time curves of AG in rats after oral administration of AG-NCs. Reprinted with permission from Ref. 182 Copyright © 2025, American Chemical Society.

Andrographolide (AG), a bioactive diterpenoid extracted from *Andrographis paniculata* used clinically against infections, suffers from low aqueous solubility and poor bioavailability^184^. Recently, Ding et al.^182^ developed AG nanocrystals (AG-NCs) with various nonionic surfactants (Pluronic-F127, TPGS, or Brij-S20) using a combination of precipitation and sonication (Fig. 15A and B). The resulting AG-NCs demonstrated a remarkable increase in the dissolution rate of AG, attaining 91.4% AG release within 5 min, which was significantly greater than the 78% and 12% release rates observed for AG microcrystals (AG-MCs) and free AG, respectively (Fig. 15C)^182^. Also, the AG-NCs exhibited superior performance in terms of cellular uptake, cumulative permeability and oral bioavailability (Fig. 15D–F)^182^. Moreover, this research demonstrates that excessive nonionic surfactants can induce the micelle formation that subsequently adheres to the AG-NCs surface and compromises particle size stability, highlighting surfactant concentration as a key criterion for nanocrystal formulation^182^.

Understanding the *in vivo* fate of drug nanocrystals is crucial for designing formulations that maximize the bioavailability of poorly soluble drugs. However, direct visualization of intact nanocrystals and their biological transformations *in vivo* remains challenging due to the limited resolution and sensitivity of conventional analytical techniques. Early studies inferred the *in vivo* fate from *in vitro* dissolution, pharmacokinetics, and biodistribution, assuming that, regardless of the administration route, nanocrystals dissolve rapidly in aqueous body fluids owing to their small particle size and then diffuse across biological barriers driven by a high concentration gradient^185^. Recent advances in bioimaging techniques, including electron microscopy, autofluorescence imaging, hybrid nanocrystals with aggregation-induced emission (AIE), aggregation-caused quenching (ACQ), and fluorescence resonance energy transfer (FRET) probes^186^, ^187^, ^188^, have enabled real-time and direct visualization of nanocrystals *in vivo*. For example, Zhang et al.^189^ constructed 2-((5-(4-(dip-tolylamino)phenyl)thiophen-2-yl)methylene)malononitrile (MeTTMN)-labeled coumarin 6 (C6) nanocrystals for simultaneous tracking of drug nanocrystals *in vivo* and dissolved free drug in body fluids. MeTTMN-labeled C6 nanocrystals can be absorbed either as in molecular form or as intact nanocrystals^189^. Similarly, quercetin hybrid nanocrystals (QT-HNCs) retained their nanocrystalline structure for up to 48 h following oral and intravenous administration in SD rats^190^. These studies demonstrate that nanocrystals are not always instantaneously dissolved and can undergo direct absorption as intact particles^185^^,^^191^. This implies that the enhanced bioavailability of nanocrystal drugs stems from multiple factors, including increased solubility and dissolution rates, improved mucoadhesion, facilitated membrane translocation, and stabilizer-mediated absorption enhancement^192^, ^193^, ^194^, ^195^.

Nanocrystals represent an effective strategy to enhance drug solubility and bioavailability of poorly water-soluble drugs, but their physicochemical stability remains a significant barrier to broader application. In addition, challenges such as poor reproducibility, batch-to-batch variability, suboptimal *in vitro*–*in vivo* correlation, and complex absorption mechanisms continue to impede clinical translation. Furthermore, the development of continuous, controlled, and robust preparation methods to produce nanocrystals with narrow particle size distributions is crucial for ensuring consistent product quality. Finally, a deeper understanding of the *in vivo* fate of nanocrystals and the establishment of predictive *in vitro*–*in vivo* models will be essential to reduce development costs and accelerate regulatory approval.

## 6 Organic framework solids

The incorporation of low water solubility drugs into porous materials has emerged as an innovative strategy to enhance drug stability and dissolution properties^196^^,^^197^. In recent decades, the rapid advancement of organic frameworks (OFs) has introduced a fascinating array of porous materials. OFs are a category of porous crystalline materials composed of organic molecules and/or ions/clusters interconnected through covalent or noncovalent bonds, featuring high surface areas and adjustable porosity. These frameworks can be categorized into two main types: covalently bonded frameworks, including metal‒organic frameworks (MOFs) and covalent organic frameworks (COFs), and noncovalently bonded frameworks, such as hydrogen-bonded frameworks (HOFs) and halogen-bonded organic frameworks (XOFs), among others^198^.

**Figure 16:**
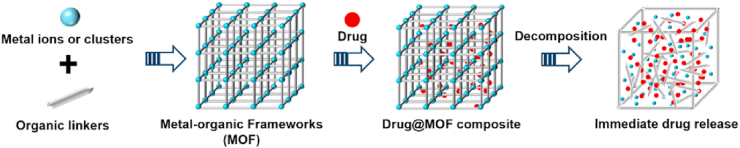
Graphical representation of the crystal engineering and drug loading of MOFs.

As novel drug delivery materials, OFs offer numerous advantages over traditional delivery systems such as liposomes, polymeric micelles, and inorganic particles. These benefits include high drug loading capacity, stability, customizable size and porosity, and ease of surface functionalization^199^, ^200^, ^201^. The highly porous nature of OFs provides exceptional potential for loading poorly water-soluble drugs, making them effective carriers for improving drug solubility and bioavailability. Among the various OFs, MOFs stand out because they are constructed from a diverse range of metal ions/clusters and organic ligands connected by weak coordination bonds (Fig. 16). This structural feature makes MOFs particularly suitable for biomedical and drug delivery applications, as they tend to degrade more readily in biological environments than other organic frameworks^202^.

The preparation of drug-loaded MOFs can be accomplished through two primary approaches: the two-step method and the one-step method^203^. The two-step strategy involves sequential processes, beginning with the synthesis of blank MOFs followed by a drug loading step. This loading can be achieved either through immersion of the synthesized MOFs in concentrated drug solutions or *via* mechanochemical grinding of drugs with preformed MOFs. Alternatively, one-step methods integrate drug molecules directly into the MOF during the synthesis process. In this approach, drugs can be incorporated either within the pore cavities of the crystal lattice or immobilized in lattice defects, with the specific location determined by the MOF pore size and the drug's molecular weight. Recent advances in MOF synthesis have led to the development of organic–ligand-free MOFs, where drug molecules serve as structural linkers themselves^204^. This innovative strategy has been exemplified by the development of Zn-curcumin MOFs, representing a significant advancement in simultaneous synthesis and drug loading methodologies^205^.

The characterization and quality control of MOFs and drug-loaded MOFs are crucial for determining their pharmaceutical performance^206^^,^^207^. For drug payload analysis, Fourier transform infrared spectroscopy (FT-IR), Raman spectroscopy, and solid-state nuclear magnetic resonance spectroscopy (ssNMR) are commonly used to investigate the molecular state of a drug and its interactions with the MOF framework. However, these conventional techniques exhibit limitations in sensitivity and resolution, particularly when analyzing MOFs with low drug loading capacities^208^^,^^209^. To address these challenges, emerging techniques such as photoluminescence spectroscopy using environment-responsive fluorophores have been developed and implemented as promising tools to overcome existing limitations and enhance characterization capabilities^189^^,^^210^, ^211^, ^212^, ^213^. These advanced methods provide additional insights and complement traditional characterization approaches in MOF analysis.

**Figure 17:**
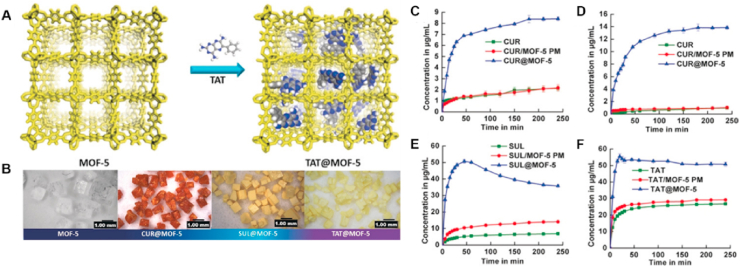
(A) Illustration of the encapsulation of the TAT drug into MOF-5 (approximately two TAT molecules per cage were encapsulated in MOF-5). (B) Optical images of MOF-5 and drug@MOF-5 composite crystals. Representative curcumin (CUR), CUR@MOF-5 and physical mixture (PM) dissolution profiles in (C) simulated gastric and (D) phosphate buffer saline media. (E) Representative sulindac (SUL), SUL@MOF-5, and SUL/MOF-5 PM dissolution profiles in simulated gastric media. (F) Representative terephthalic acid (TAT), TAT@MOF-5 and TAT/MOF-5 PM dissolution profiles in phosphate buffer saline media. Reprinted with permission from Ref. 217 Copyright © 2019, Wiley.

MOFs have demonstrated significant potential in enhancing the phase stability, solubility, and bioavailability of poorly water-soluble drugs^214^^,^^215^. The incorporation of amorphous drugs into MOFs can inhibit crystallization and maintain the drugs in the amorphous state, thereby increasing their solubility. Simultaneously, MOFs optimize the dissolution of poorly water-soluble drugs by increasing the effective surface area of encapsulated drugs, analogous to particle size reduction techniques^13^^,^^216^^,^^217^. For example, Suresh and Matzger developed a water-sensitive MOF-5 constructed from zinc clusters and terephthalic acid to improve the low water solubility drugs curcumin, sulindac, and triamterene^217^. As shown in Fig. 17A and B, a post synthetic drug loading strategy was employed, in which activated MOF-5 crystals were immersed in concentrated drug solutions, resulting in molecular encapsulation of the APIs in their amorphous state. The drug@MOF-5 composite system effectively prevented recrystallization of the amorphous drugs (stable for >4 months) and facilitated immediate drug release in dissolution media upon hydrolytic MOF decomposition^217^. The maximum concentrations of drugs (curcumin, sulindac and terephthalic acid) released by MOF-5 showed significant enhancement compared to their neat crystalline forms (Fig. 17C–F)^217^.

**Figure 18:**
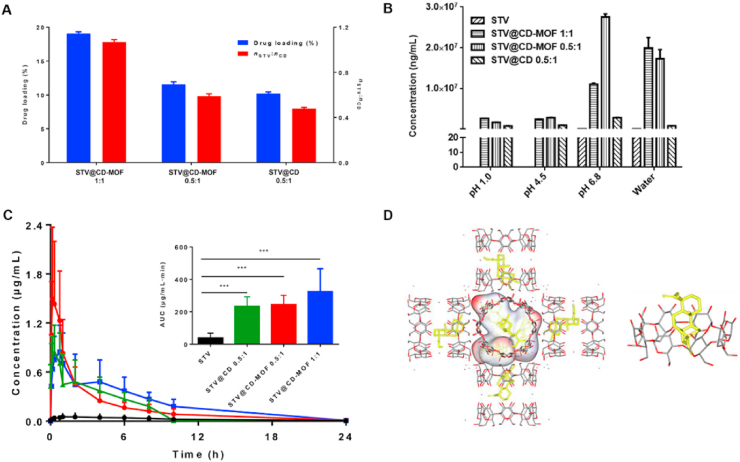
Solubility and bioavailability improvement of isosteviol (STV) using cyclodextrin metal–organic frameworks. (A) Efficient loading of the STV by the CD-MOF. (B) Compared with that of STV and STV@CD, the solubility of STV@CD-MOF was significantly greater at different pH values. (C) Plasma concentration‒time profiles of the STV in rats after oral administration of STV@CD-MOF (1:1). **(**D**)** Molecular mechanism of the STV distribution in CD-MOF as a nanocluster and CD complexation. Reprinted with permission from Ref. 216 Copyright © 2021, Elsevier.

Isosteviol (STV), a synthetic diterpene steviol glycoside derivative, has diverse biological activities and pharmacological effects, including antifungal and antineoplastic antiviral effects, improved insulin sensitivity, and reduced plasma triglyceride levels. However, the extremely low aqueous solubility of STV (only 129.58 ng/mL in water) significantly limits its bioavailability. Chen et al.^216^ utilized cyclodextrin metal–organic frameworks (CD-MOFs) to simultaneously improve the solubility and bioavailability of STV through the synergistic combination of *γ*-cyclodextrin's host–guest molecular recognition properties with the tunable porosity of metal–organic frameworks (Fig. 18A and D). Compared to free STV, the solubility of the STV@CD-MOF exhibited a dramatic increase across different dissolution media (Fig. 18B). Furthermore, following oral administration in rats, the AUC_0–24h_ for STV@CD-MOFs was 8.67 times greater than that of free STV (*P* < 0.001) (Fig. 18C)^216^.

**Figure 19:**
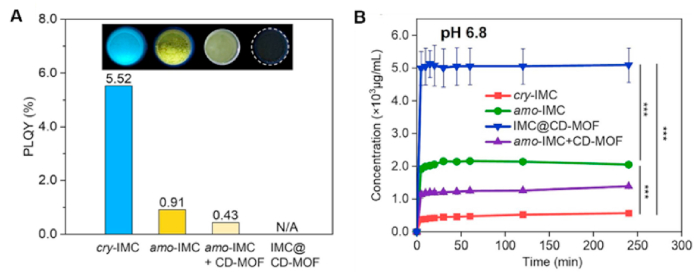
The inherent AIE feature revealed the drug molecular state in CD-MOFs for enhanced dissolution. (A) PLQY and fluorescence microscopy images of different IMC solid forms. (B) *In vitro* dissolution profiles of cry-IMC, amo-IMC, IMC@CD-MOF, and amo-IMC + CD-MOF in pH 6.8. Reprinted with permission from Ref. 12 Copyright © 2023, Elsevier.

Utilizing a nanoconfinement strategy, amorphous indomethacin (IMC, a nonsteroidal anti-inflammatory drug) was stabilized within a porous *γ*-cyclodextrin-based metal–organic framework (CD-MOF)^13^. The aggregation-induced emission (AIE) property of IMC facilitated sensitive fluorescence imaging, which confirmed its high-energy, non-aggregated state inside CD-MOF through AIE turn-off (Fig. 19A). IMC@CD-MOF demonstrated high stability over 100 days under accelerated conditions (60 °C/75% RH). Dissolution profiles revealed exceptionally rapid release, with the highest drug concentration achieved within 5 min across diverse media (pH 1.2, 4.5, 6.8 buffer and deionized water). In a pH 6.8 buffer, the maximum concentration (*C*_max_) of IMC@CD-MOF reached approximately 5 mg/mL, which was a 10-fold and 5-fold increase compared to crystalline and amorphous IMC, respectively^13^ (Fig. 19B).

Through rational crystal engineering and optimized drug loading, MOF-based delivery systems offer multidimensional solutions for stabilizing, solubilizing, and delivering challenging drug molecules. However, several critical challenges must be addressed to bridge the gap from “proof-of-concept” to clinical utility, including biostability, biosafety, biopharmaceutical manufacturing, and quality control of MOF-based formulations. For example, achieving a balance between the degradability and stability of MOFs is essential to ensure precision drug release in complex biological or physiological environments. Additionally, a comprehensive evaluation of the long-term *in vivo* toxicity of MOF-based drug delivery systems (DDSs), such as metal ion accumulation and the toxicity of degradation products, is crucial, as these factors determine their eligibility for clinical studies. The use of biocompatible organic linkers and alkali metal cations (*e.g.*, Na^+^, K^+^), which are essential mineral elements in the human body, in MOF fabrication can significantly increase their clinical translation potential^218^. Future advancements in this field should integrate computational materials science, precision medicine, and smart manufacturing to enable industrial-scale production and rigorous quality control of MOF-based drug delivery systems. Such interdisciplinary efforts will be pivotal in overcoming existing challenges and realizing the full potential of MOFs in pharmaceutical applications.

## 7 Solid solutions

**Figure 20:**
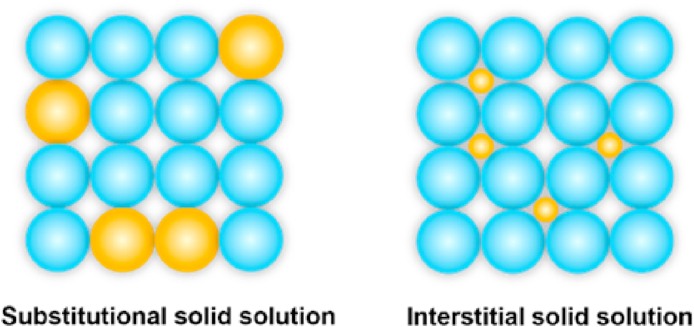
Illustration of common types of solid solutions.

A solid solution is a homogeneous crystalline phase in which two or more components are uniformly dispersed at the atomic or molecular level while preserving the crystal structure of the host material^219^^,^^220^. This phenomenon usually occurs when the components share similar crystal structures, atomic sizes, and chemical characteristics, which enables the solute atoms to be incorporated into the host lattice either substitutionally (replacing atoms in the host lattice) or interstitially (filling the interstitial sites within the host lattice), as shown in Fig. 20. Typically, solid solutions can exhibit a continuous composition range, extending from trace incorporation of the guest component at undetectable molar fractions (xg ≈ 0) through equimolar stoichiometry (xg = 0.5) to compositions where the guest component becomes predominant (xg > 0.5), effectively reversing the host‒guest relationship within the crystalline matrix^221^. Therefore, compared with salts and cocrystals, which have fixed stoichiometric ratios, solid solutions can achieve continuous variation in the stoichiometric ratios between components.

Molecular size and shape similarity have long been recognized as significant factors in solid solution formation and mutual miscibility of components in the solid state constitutes a fundamental requirement for this process, dependent on structural mimicry and crystal isomorphism^222^. Moreover, weak intermolecular forces represent a critically important factor regulating crystal structure stability and thereby influencing molecular solid solution formation^223^. While, it is reported that solid solutions between similar molecules can form if substituted atoms or groups do not participate in significant directional or electrostatic interactions^224^. Therefore, further work is necessary to establish predictive principles for solid solution formation across diverse molecular systems.

**Figure 21:**
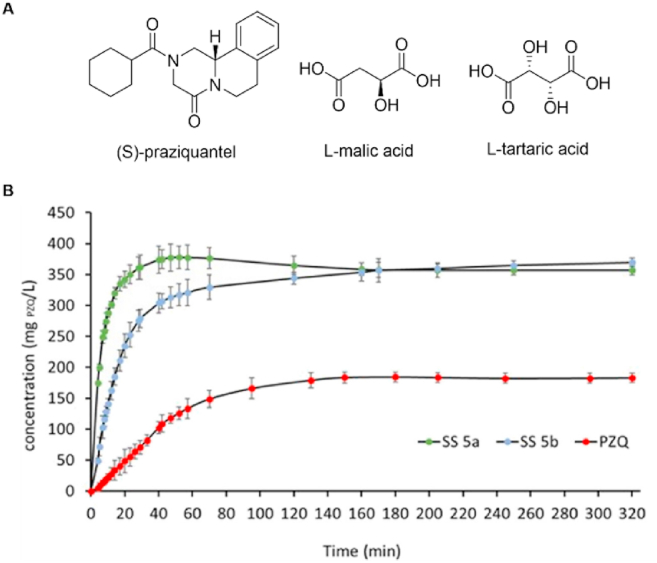
(A) The chemical structure of *S*-praziquantel, L-malic acid and L-tartaric acid. (B) *In vitro* dissolution kinetics of solid solutions SS 5a and SS 5b and pure praziquantel (PZQ) in water. Reprinted with permission from Ref. 13 Copyright © 2023, The Authors.

Solid solutions have been applied to improve the physicochemical properties of APIs, such as hardness^225^, melting point ^226^, dissolution and bioavailability of poorly water-soluble drugs^14^. For example, Desiraju's group^225^ modulated the hardness of omeprazole by systematically varying the proportions of the 5- and 6-methoxy tautomers of omeprazole. This improvement in hardness is attributed to the increased resistance of the lattice to shear sliding of molecular layers under plastic deformation. Furthermore, the formation of a solid solution can affect the physical stability of polymorphs^227^. Benzamide exists in multiple polymorphic forms, among which Forms II and III are particularly difficult to crystallize. A recent study demonstrated that benzamide Form I readily converts to Form III *via* mechanochemistry in the presence of nicotinamide, driven by a thermodynamic switch and the formation of solid solutions, enabling the robust and exclusive crystallization of the otherwise elusive Form III^220^. Praziquantel (PZQ), which belongs to the class II category of chiral drugs, is employed as a racemate in the treatment of schistosomiasis. As shown in Fig. 21, Cappuccino et al.^14^ developed a series of solid solutions of praziquantel with enantiomers of malic acid and tartaric acid and reported that the solid solutions of SS 5a (*R*-praziquantel/*S*-praziquantel/l-malic acid/l-tartaric acid = 1:1:1:1) and SS 5b (*R*-praziquantel/*S*-praziquantel/l-malic acid/d-tartaric acid = 1:1:1:1) provided a highly soluble and rapidly absorbing form of praziquantel. Furthermore, a solid solution combining cortisone and cortisol (hydrocortisone) as a drug‒prodrug system exhibited a dissolution rate nearly double that of pure hydrocortisone^219^.

Deep eutectic solvents (DESs) are liquids formed by two or more components associating through hydrogen bonding, which depresses the melting point sufficiently to remain a liquid state at room temperature^228^^,^^229^. In recent years, DESs have emerged as promising candidates for oral drug delivery formulations due to their exceptional solubilization capacity for poorly soluble drugs^230^^,^^231^. For example, DESs enhanced the solubility of aprepitant^232^, indomethacin^233^, and venetoclax^234^ by up to 1057-, 159,000-, and 118,200-fold, respectively, compared to their aqueous solubility. Additionally, API-based DES systems (termed as therapeutic deep eutectic solvents) represent a viable approach for developing liquid pharmaceutical formulations, where APIs act as hydrogen bond donors and/or acceptors to form DESs with other components^235^^,^^236^. For example, Panbachi et al.^230^ developed fixed-dose combinations of abiraterone acetate using ibuprofen-based DESs. *In vitro* dissolution studies demonstrated that these drug-based DESs increased drug release, achieving 37.8 ± 9.0% to 64.2 ± 1.0% for ibuprofen and 5.0 ± 3.3% to 19.4 ± 0.1% for abiraterone acetate. Furthermore, DESs also function as novel transdermal delivery vehicles by enhancing drug solubility, opening tight junctions in the stratum corneum, modifying keratin conformations, and facilitating lipid exchange^237^. For instance, the DES formation of ketoprofen and flurbiprofen with lidocaine increased transdermal flux by 3.8-and 4.7-fold, respectively, compared to simple saturated aqueous solutions^238^. However, several critical challenges must be addressed for successful clinical translation of DESs^239^^,^^240^. Studies indicate increased toxicity in API-DES systems relative to individual components^241^^,^^242^, necessitating rigorous toxicity profiling. The functional mechanisms of APIs within eutectic systems remain incompletely elucidated and warrant further investigation. Additionally, it remains unclear whether biological activities originate from individual drugs or emerge as collective properties of the eutectic mixture itself. Regarding regulatory considerations, while current research suggests generally mild toxicity of DESs in biological environments in current research, their clinical translation requires strict alignment with international pharmaceutical standards and governmental regulations.

## 8 Liquid crystals

**Figure 22:**
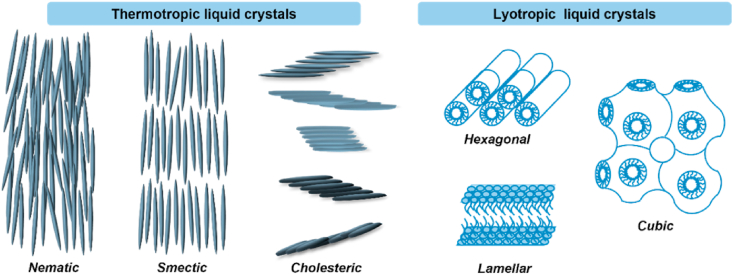
Schematic diagram of LCs.

The first liquid crystal, cholesteryl benzoate, was discovered by the Australian botanist Freidrich Reinitez in 1888^243^. With development and exploration over a century, liquid crystals have been widely used in a variety of fields, such as chemistry, physics, biology, and electronic engineering^244^. In the biomedicine field, liquid crystal technology has been applied in drug delivery, bioimaging, tissue engineering, implantable devices, biosensing and wearable devices^245^. Liquid crystals (LCs) are defined as a state of matter that exists in an intermediate position between the solid and liquid states, featuring anisotropic properties such as a crystalline solid, coupled with the fluidity of the liquid state^246^. LCs can be subdivided into two classes on the basis of their formation mechanism: thermotropic liquid crystals (TLCs) and lyotropic liquid crystals (LLCs), as shown in Fig. 22 ^246^.

**Figure 23:**
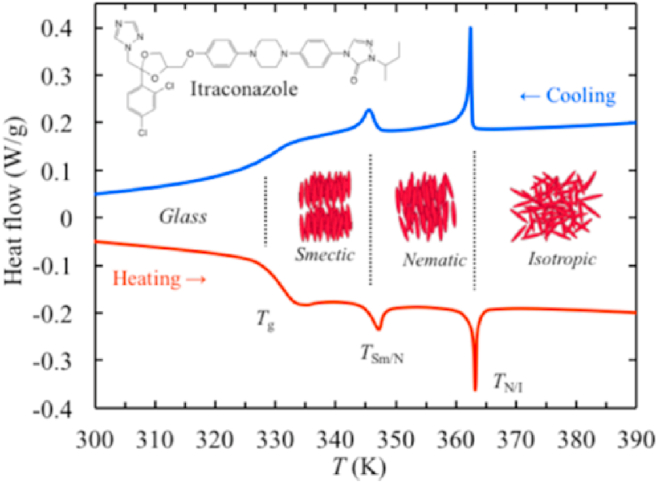
Differential scanning calorimetry (DSC) traces of itraconazole during cooling and reheating at 0.16 K/s. Reprinted with permission from Ref. 248 Copyright © 2018, the American Physical Society.

TLCs are formed by the interplay of anisotropic molecules, which can be further classified into nematic, smectic and cholesteric phases^246^^,^^247^. Mesophase transitions, driven by variations in temperature, are usually fast and easily reversible^248^. Traditional theories of these transitions do not include time as a variable, assuming phase changes to be instantaneous and controlled by thermodynamics rather than kinetics^249^, ^250^, ^251^. However, recent studies have shown that these transitions can be frustrated and even eliminated by applying a moderate cooling rate^248^^,^^252^^,^^253^. For example, in the antifungal drugs itraconazole and saperconazole, smectic orders (including no order) can be selectively accessed by cooling the isotropic liquid at different rates, providing a reference for designing liquid crystals with tunable order tailored to specific applications^248^^,^^252^^,^^253^. LLCs, on the other hand, are formed by the self-assembly of amphiphilic molecules under specific conditions (solvent, concentration, temperature, pH, etc.)^254^^,^^255^. LLCs include lamellar, hexagonal, cubic mesophases, among others^255^^,^^256^. Several small-molecule drugs exhibit LC behavior, including TLCs (*e.g.*, itraconazole (Fig. 23), saperconazole, methotrexate, fenoprofen, nafoxidine hydrochloride, cyclosporine) and LLCs (*e.g.*, fenoprofen calcium, ketoprofen, nafoxidine hydrochloride, nafcillin sodium, diclofenac, flufenamic acid, and tobramycin)^248^^,^^252^^,^^257^^,^^258^. Owing to their variation in spatial organization and free Gibbs energy, LCs have the potential to improve the dissolution and bioavailability of poorly water-soluble drugs. Furthermore, the tunable nanostructures and dynamic behavior of LCs offer versatile platforms for drug delivery applications to achieve sustained release, enhanced solubility, and targeted delivery of poorly water-soluble drugs^259^^,^^260^.

While TLCs have seen limited exploration as drug carriers, LLCs have been extensively developed for drug delivery systems *via* various routes (*e.g.*, intranasal, oral, pulmonary, ocular, and vaginal) due to their concentration-dependent liquid crystallinity^259^, ^260^, ^261^, ^262^, ^263^, ^264^, ^265^, ^266^. In addition, the viscoelastic nature of LLCs enables their application in dispersion technology. A highly ordered, thermodynamically stable internal nanostructure makes it a potential delivery matrix for sustained release^267^^,^^268^. The *in vitro* and *in vivo* release profiles of LLCs are related to their structure, particle size, drug properties, and *in vivo* fate^269^, ^270^, ^271^, ^272^. Using LCs as oral carriers for poorly water-soluble drugs provides key advantages: stabilizing drugs *via* isolation from the physiological environment, promoting adhesion to biological membranes, and minimizing the impact of GI tract water fluctuations^273^, ^274^, ^275^.

**Figure 24:**
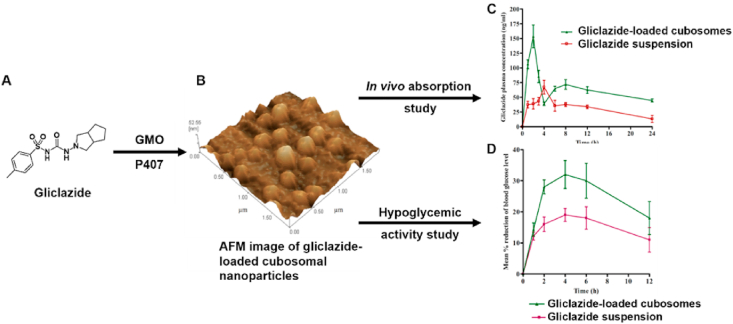
Bioavailability and antidiabetic activity of gliclazide-loaded cubosomal nanoparticles. (A) Chemical structures of gliclazide. (B) AFM photomicrograph of gliclazide-loaded cubic liquid crystalline. (C) Plasma concentration–time profiles of gliclazide following a single oral dose (10 mg/kg) of gliclazide formulations in rats. (D) Comparative evaluation of hypoglycemic activity after administration of the different gliclazide formulations. Reprinted with permission from Ref. 14 Copyright © 2021, The Authors.

Gliclazide (GLZ) is a hypoglycemic drug used for the treatment of noninsulin-dependent diabetes mellitus (Fig. 24A)^15^. As a BCS Class II drug, GLZ has a slow absorption rate and poor bioavailability^276^. Nasr et al.^15^ prepared a GLZ-loaded cubic liquid crystalline phase using glyceryl monooleate (GMO) as a forming material (Fig. 24B). Compared with the GLZ suspension, the LCs exhibited accelerated *in vitro* release behavior^15^. Furthermore, pharmacokinetic studies following oral administration to rats revealed a greater plasma concentration at nearly all time points over 24 h, resulting in a 2-fold increase in the bioavailability of the GLZ LCs relative to the GLZ suspension (Fig. 24C)^15^. In terms of hypoglycemic activity, GLZ LCs presented superior performance over 12 h, as evidenced by a more pronounced reduction in blood glucose levels (Fig. 24D)^15^.

**Figure 25:**
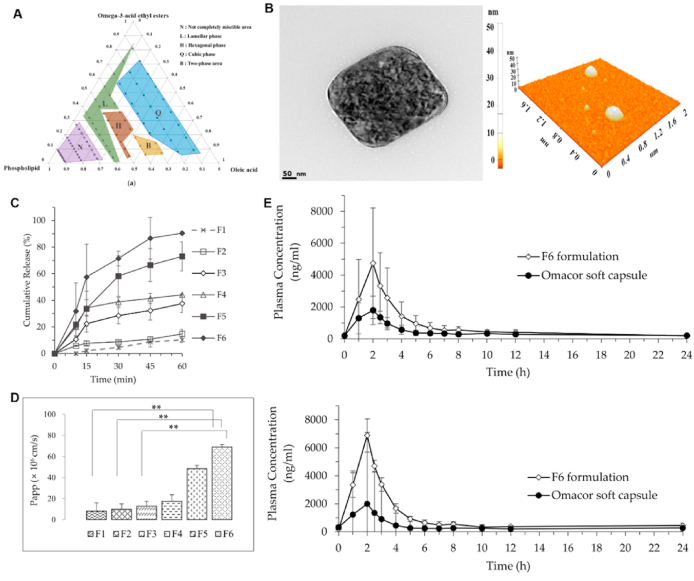
Formation of self-assembled liquid crystalline nanoparticles and absorption enhancement of Ω-3s by phospholipids and oleic acids. (A) Ternary phase diagram of Ω-3-acid ethyl esters (Ω-3 EE), oleic acid, and phospholipids in water at 25 °C. (B) Morphological characterization of LCs using TEM (left) and AFM (right). (C) *In vitro* dissolution of the DHA in the biomimetic test solution. (D) Apparent permeation coefficient for DHA, (E) Plasma concentration–time profiles of LCs formulations (up: EPA, down: DHA) and Omacor soft capsules after oral administration of a 1000 mg single-dose of Ω-3 EE in male beagle dogs. F1 formulation did not form LCs; F2 formulation formed a lamellar LC; F3 formulation formed a hexagonal LC; and F4, F5, and F6 formulations formed cubic LCs. Reprinted with permission from Ref. 278 Copyright © 2021, The Authors.

The *n*-3 polyunsaturated fatty acids (Ω-3s) are essential fatty acids, the ethyl esters of which are approved by the FDA for treating hypertriglyceridemia in most cardiovascular diseases. However, the low solubility in the gastrointestinal (GI) tract and poor stability of Ω-3s ethyl esters lead to low bioavailability^277^. Jeon and coworkers^278^ prepared multiple liquid crystals loaded with Ω-3s ethyl esters by varying the formulation composition through a ternary phase diagram (Fig. 25A and B). The dissolution and permeation results revealed that cubic liquid crystals performed better than lamellar and hexagonal liquid crystals because of their ability to spontaneously form nanoparticles with smaller particle sizes in the GI tract-like test solution (Fig. 25C and D)^278^. As a result, cubic liquid crystals of Ω-3s ethyl esters were selected for pharmacokinetic studies in comparison with Omacor soft capsules in beagles. The bioavailabilities of both LC-loaded eicosapentaenoic acid (EPA) and docosahexaenoic acid (DHA) increased, with 2.5- and 3.1-fold higher pharmacokinetic parameters AUC_all_ than Omacor soft capsule, respectively (Fig. 25E)^278^.

LCs offer a promising approach to enhance the bioavailability of poorly soluble drugs. However, their clinical translation remains challenging, evidenced by the limited number of marketed products. Formulation design needs to balance high drug loading with liquid crystal phase stability, as factors like temperature, pH, and shear stress can induce phase transitions that alter drug release behavior. Detailed structural characterization and monitoring of liquid crystal behavior in biological environments remain inadequate, hindering a complete understanding of their *in vivo* fate. Future studies would benefit from integrating molecular modeling and artificial intelligence to predict liquid crystal properties and streamline the selection of optimal formulations^279^. Furthermore, developing advanced analytical and imaging techniques to characterize mesophase architecture and track formulation behavior *in vivo* will provide the mechanistic insights required to rationally optimize release performance and accelerate clinical translation.

## 9 Amorphous solids

Amorphous solids, characterized by their disordered molecular arrangement, are widely used to improve the dissolution rate and apparent solubility of poorly water-soluble drugs^16^^,^^280^, ^281^, ^282^, ^283^. However, due to the thermodynamic instability of amorphous systems, they exhibit a strong tendency toward crystallization. A variety of stabilization strategies have been proposed, including polymer-based amorphous solid dispersion (ASD)^16^, coamorphous formulation^284^, and mesoporous material-based drug delivery systems^285^.

ASD refers to a system in which the API is molecularly dispersed in an amorphous state within a polymer matrix^16^, which can effectively inhibit the crystallization of amorphous drugs. Sorafenib (SOR), a BCS Class II drug used to treat hepatocellular carcinoma, exhibits extremely poor aqueous solubility (∼10 ng/mL in 50 mmol/L phosphate-buffered saline (PBS, pH 6.8) at room temperature)^286^. To address this limitation, Song et al.^286^ developed a 40% drug-loaded ASD of SOR using hydroxypropyl methylcellulose acetate succinate (HPMC-AS) *via* the coprecipitation method. The resulting SOR ASD tablets demonstrated approximately 50% higher relative bioavailability in dogs compared to the commercial product Nexavar®^286^.

**Figure 26:**
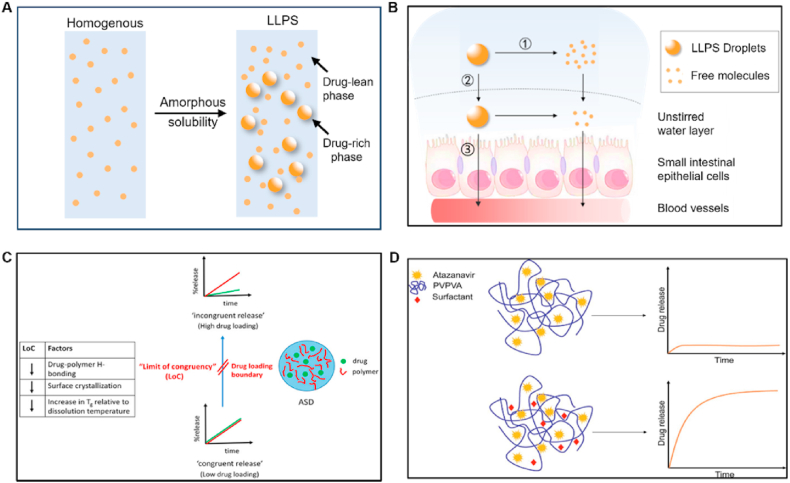
Schematic illustration of (A) liquid–liquid phase separation. (B) Supersaturation maintenance, unstirred water layer permeation mediated and direct uptake by small intestinal epithelial cells *via* droplets. (C) The dissolution model based on the limit of congruency of ASD (Adapted from Ref. 293 with permission. Copyright © 2020 by American Chemical Society). (D) Effect of surfactant on dissolution of atazanavir ASD (Reprinted with permission from Ref. 295 Copyright © 2022, Elsevier).

Recent studies have shown that liquid‒liquid phase separation (LLPS) plays a crucial role in enhancing the oral absorption of poorly water-soluble drugs. This phenomenon occurs when drug concentration exceeds its amorphous solubility, leading to the formation of drug-rich and drug-lean phases (Fig. 26A). The benefits of LLPS during ASD dissolution include (Fig. 26B): (1) drug-rich droplets serve as reservoirs, continuously releasing drug molecules to maintain supersaturation in the aqueous phase, thereby compensating for drug absorption across the gastrointestinal epithelium^287^^,^^288^; (2) drug-rich droplets reduce diffusion resistance in the unstirred water layer of the gastrointestinal tract, improving API permeability^289^^,^^290^; (3) drug-rich droplets may be directly absorbed by small intestinal epithelial cells^291^.

Additionally, the congruent release of drug and polymer is important for inducing LLPS^292^. As shown in Fig. 26C, the limit of congruency (LoC) is a key drug loading threshold governing the release behavior of the API and polymer. Below the LoC threshold, both components exhibit rapid and complete congruent release^293^^,^^294^. However, when drug loading exceeds the LoC, amorphous–amorphous phase separation (AAPS) occurs in the ASD system, leading to the formation of drug-rich interfacial domains and subsequently incongruent release of API and polymer, effectively suppressing LLPS formation^294^. The incorporation of surfactants into the ASD has shown promise in improving the LoC^295^^,^^296^. For example, Correa-Soto et al.^295^ demonstrated that surfactants significantly increased the LoC in atazanavir-PVP/VA ASDs (Fig. 26D). Adding 5% *w*/*w* of either sodium dodecyl sulfate (SDS) or cetrimonium bromide (CTAB) to the binary ASD doubled the LoC (from 5% to 10% drug loading), resulting in a >30-fold increase in total drug release compared to the surfactant-free system^295^. Notably, incorporation of 5% Span 80 further raised the LoC to 15% drug loading^295^.

**Figure 27:**
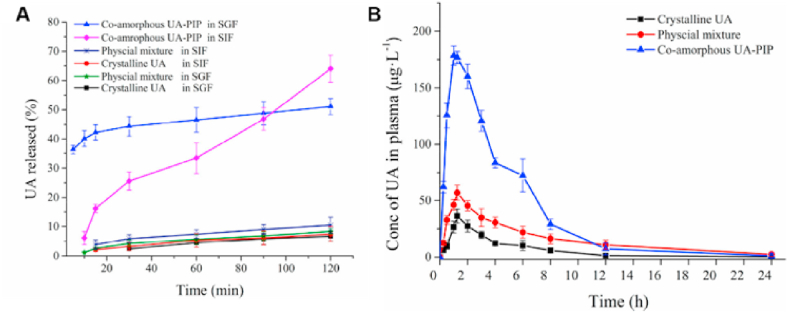
Increased dissolution (A) and oral bioavailability (B) of UA by coamorphous with PIP. Reprinted with permission from Ref. 299 Copyright © 2020, American Chemical Society.

Coamorphous binary systems, formed between a drug and a small-molecule coformer, offer a promising approach for improving drug stability and solubility^297^. The coamorphous formation with API could decrease the molecular mobility and increase the configurational entropy of the amorphous API, which is beneficial for hindering its crystallization^298^. Ursolic acid (UA), classified as a BCS IV compound, has limited oral bioavailability due to poor aqueous solubility and low intestinal permeability^299^. Yu et al.^299^ prepared a coamorphous system of UA with piperine (PIP) to increase the solubility and permeability of UA. Compared with crystalline UA and the physical mixture, the coamorphous system demonstrated a 5.3- to 7-fold increase in solubility (Fig. 27A). Furthermore, pharmacokinetic studies in rats revealed that the coamorphous UA formulation significantly enhanced oral exposure, with AUC_0–∞_ values 5.8- and 2.47-fold greater than those of crystalline UA and the physical mixture, respectively (Fig. 27B). *In vitro* permeability studies using Caco-2 cell monolayers and metabolism assays with rat hepatic microsomes demonstrated that free PIP significantly enhanced UA permeability while effectively inhibiting the CYP3A4-mediated enzymatic metabolism of UA^299^.

**Figure 28:**
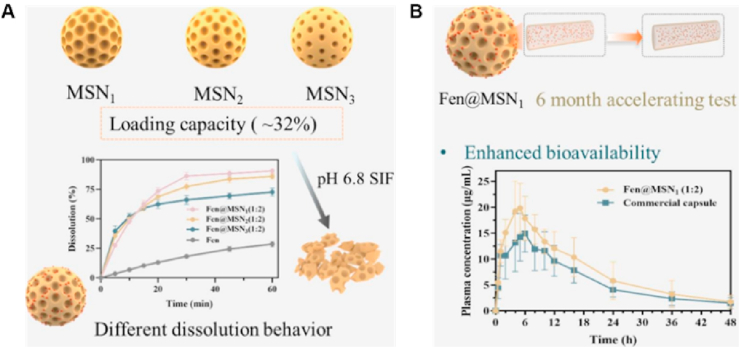
Variable pore size of mesoporous silica in improving physical stability, dissolution and oral bioavailability of fenofibrate (Fen). (A) The solubilization effect of mesoporous silica on the insoluble drug Fen and (B) enhanced oral bioavailability. Reprinted with permission from Ref. 302 Copyright © 2025, Elsevier.

In mesoporous materials systems, APIs are either adsorbed onto pore surfaces or physically confined within mesopores. The confinement of APIs within the porous architecture and the size constraint effects of mesopores could effectively stabilize amorphous drugs^300^^,^^301^. Qi et al.^302^ evaluated the effects of the mesoporous silica pore size on improving the physical stability and oral bioavailability of the insoluble drug fenofibrate (Fen). The study demonstrated that the drug release rate from mesoporous silica significantly increased with increasing pore size. Notably, the drug-loaded mesoporous silica achieved 95% release within 1 h, in contrast to pure crystalline Fen, which exhibited only 25% dissolution over the same period (Fig. 28A)^302^. Furthermore, as shown in Fig. 28B, pharmacokinetic evaluations in rats revealed that the mesoporous silica-based drug delivery system exhibited a 1.3-fold greater oral bioavailability than the commercial capsule (Lipanthyl®)^302^. These findings highlight mesoporous silica carriers as highly promising nanomaterials with substantial potential for pharmaceutical applications in enhancing low water solubility drugs.

**Figure 29:**
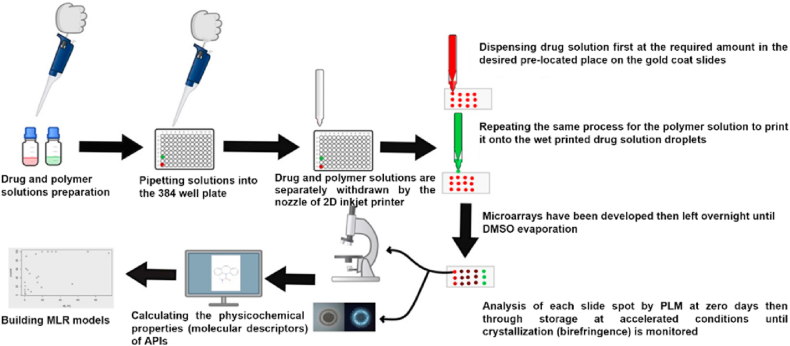
Schematic representation of the steps followed in the manufacturing and analysis of microarrays. Reprinted with permission from Ref. 310 Copyright © 2024, The Authors.

Currently, the formulation design of amorphous pharmaceuticals still largely relies on trial‒and‒error approaches, which require extensive dissolution and stability testing. This empirical process not only consumes time and resources but also slows the overall development cycle. Recently, several mathematical models and machine learning methods have been developed to predict the properties of amorphous solids, such as physical stability, dissolution performance, oral absorption and shelf-life^303^, ^304^, ^305^, ^306^, ^307^, ^308^. For example, Chu Leon et al.^309^ developed an accelerated stability assessment program (ASAP) for griseofulvin/HPMC-AS ASDs, employing an isoconversion method (time to reach specification limits) combined with a modified Arrhenius approach. This methodology enables reliable shelf-life predictions from short-term stability data^309^. Ghazi et al.^310^ implemented a high-throughput microarray system to evaluate ASD physical stability under accelerated conditions (40 °C/75% RH) over 6 months (Fig. 29). Using multiple linear regression (MLR), the authors identified the key API characteristics influencing stability and reported that a lower amount of hydrogen bond acceptors and a greater total number of heteroatoms and oxygen atoms of the API, as well as a lower melting point of the API, were beneficial for improving the stability of the ASD^310^. Furthermore, Han et al.^311^ constructed a comprehensive machine learning framework for ASD stability prediction, incorporating 646 stability data points, over 20 molecular descriptors, processing and storage parameters. The random forest (RF) model demonstrated superior predictive accuracy (82.5%)^311^. The integration of experimental data with computational modeling shows promise for accelerating formulation development while reducing resource requirements.

**Figure 30:**
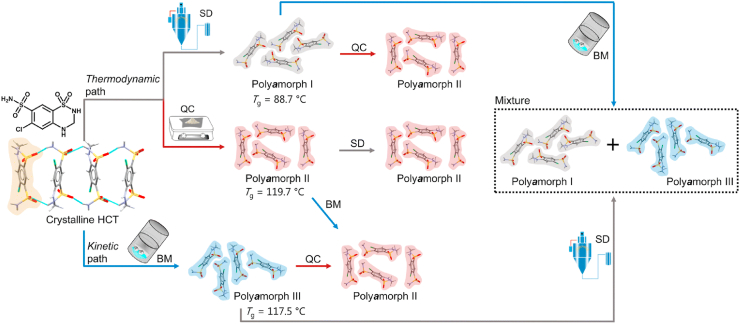
Schematic representation of the formation of HCT polyamorphs and their respective polyamorphic interconversions *via* both thermodynamic and kinetic pathways. Reprinted with permission from Ref. 318 Copyright © 2023, The Royal Society of Chemistry.

Various methods have been employed to prepare amorphous solids, including hot-melt extrusion, spray drying, milling, electrospinning, 3D-printing and co-precipitation^16^^,^^312^^,^^313^.The preparation method can significantly influence the physical stability and dissolution performance of amorphous solids^314^^,^^315^. For instance, Wolbert et al.^315^ demonstrated that griseofulvin ASDs produced *via* hot-melt extrusion, typically yielding larger particle sizes, exhibited greater physical stability than the spray-dried product with smaller particle sizes. Recent studies have shown that preparation techniques can induce distinct structural variations in amorphous materials, leading to marked differences in their physicochemical properties^316^, ^317^, ^318^, ^319^. For example, different amorphous forms of hydrochlorothiazide (HCT), were prepared *via* spray drying (SD, polyamorph I), quench cooling (QC, polyamorph II) and ball milling (BM, polyamorph III), followed by evaluation of their polyamorphic interconversions^318^. As illustrated in Fig. 30, these amorphous forms exhibited varying *T*_g_ values, relaxation time and physical stabilities. Molecular simulations further revealed significant differences in their dihedral angle distributions of HCT molecules in polyamorphs I and II^318^. These findings highlight the critical role of processing method selection in optimizing the performance of amorphous pharmaceutical formulations.

Overcoming the fundamental unpredictability of crystallization kinetics across diverse conditions represents a paramount challenge in amorphous drug development^320^. Although nucleation and growth mechanisms are well understood, a priori prediction remains elusive, critically undermining formulation stability assurance. Compounding this, the extreme sensitivity of supersaturated solution stability during dissolution to numerous factors creates major barriers to establishing reliable *in vitro*–*in vivo* correlations, demanding urgent research into optimized biorelevant testing methods. Furthermore, the essential formulation trade-off, where sufficient polymer use ensures stability but causes low drug loading and patient-unfriendly dosing, directly impacts therapeutic utility and commercial viability. Emerging surfactant-containing formulations offer drug-loading improvements, while the impact of surfactants type, amount and incorporation method on processing, dissolution performance, and ultimately *in vitro*–*in vivo* correlation remains poorly understood. While most research on amorphous drugs has primarily focused on immediate-release formulations, supersaturation, and stability, sustained-release systems based on amorphous formulations are increasingly being interested for poorly water-soluble drugs with short half-lives^321^. This approach uniquely merges solubility enhancement with controlled release, addressing bioavailability limitations while optimizing therapeutic efficacy and patient adherence. However, key parameters controlling both the release profile and physical stability of these sustained-release systems, such as matrix type, polymer gelling behavior, and amorphous drug tablet formulation, remain largely unexplored. Simultaneously, the rational selection of coformers and mesoporous carriers demands deeper mechanistic insights into their stabilization effects, drug distribution patterns within confined spaces, and modulation of drug release. Addressing these challenges is essential for translating the therapeutic potential of amorphous systems into reliably effective medicines.

## 10 Salts

Pharmaceutical salts are defined as compounds consisting of an ionized API and an organic or inorganic counterions^17^^,^^322^. Due to the relatively simple preparation and crystallization processes, as along with the reliability of the resulting product, salt formation is a widely used strategy for improving the key properties of drugs, including solubility, dissolution rate, stability, toxicity, mechanical properties, and scalability^17^^,^^323^, ^324^, ^325^. Notably, nearly 40% of drugs approved by the FDA from 1939 to 2023 were formulated as salts, underscoring the critical importance of this approach in pharmaceutical development^322^^,^^325^^,^^326^.

The selection of counterions for pharmaceutical salt formation is paramount, with safety serving as the primary determinant. Generally Recognized As Safe-listed counterions are most frequently employed due to their established safety profiles. Current classification systems further categorize these counterions based on origin and toxicological acceptability^322^^,^^327^: (1) Class I includes naturally occurring counterions approved for unrestricted used, such as chloride, sodium, acetate; (2) Class II comprises counterions exhibiting low toxicity and good tolerability, such as benzoate, pamoate, mesylate; and (3) Class III contains counterions with limited applications, such as bromide, nitrate, camsylate. These classifications provide valuable guidance for counterion selection. Meanwhile, critical physicochemical parameters of counterions, such as p*K*a, molecular weight and solubility must also be assessed as they directly influence both the properties of the resulting drug products and the salt formation process. For instance, Pereira et al.^328^ prepared eight carvedilol salts to enhance its poor aqueous solubility. Among these, the malonate salt exhibited a 25-fold increase in solubility, while the benzoate salt showed minimal improvement compared to the parent drug. The authors attributed the enhanced solubility to the longer and bulky polar counterions, which further separates carvedilol molecules in the crystal lattice^328^. Similarly, Gwak et al.^329^ reported three piroxicam salts formed with monoethanolamine (PRX-MEA), diethanolamine (PRX-DEA), and triethanolamine (TEA). The dissolution and pharmacokinetic behavior of these salts depended on the alkyl chain length, following the order PRX-MEA > PRX-DEA > PRX-TEA^329^. These findings highlight the importance of rational counterion selection and provide a systematic strategy for modulating salt properties. Notably, this rational selection process has been further enhanced by recent advances in machine learning, which enable prediction of drug p*K*a values, protonation state distributions, and structural properties of salt, thereby accelerating counterion selection for salt development^330^, ^331^, ^332^, ^333^. *Epik version 7*, software that employs an ensemble of atomic graph convolutional neural networks trained on >42,000 p*K*a values, demonstrates accurate prediction capabilities, with a median absolute and root mean square errors of 0.42 and 0.72 p*K*_a_ units, respectively^330^. By rapidly identifying the critical p*K*a values of compounds, such *in silico* tools guide counterion selection and focus experimental efforts on the most promising drug-counterion pairs^330^.

**Figure 31:**
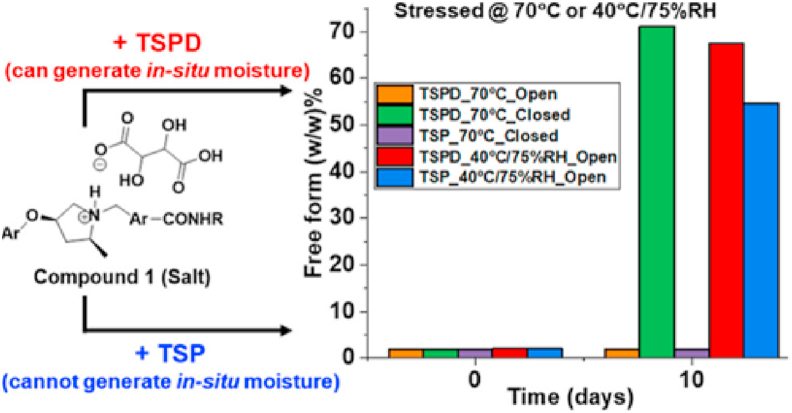
The effect of *in*-*situ*-generated moisture on the disproportionation of a pharmaceutical salt. TSPD and TSP represent trisodium phosphate dodecahydrate and trisodium phosphate, respectively. Reprinted with permission from Ref. 346 Copyright © 2023, American Chemical Society.

Meanwhile, the solid-state forms of pharmaceutical salts (*e.g.*, polymorphs^334^^,^^335^, amorphous^336^^,^^337^, hydrates/solvates^338^^,^^339^ and cocrystals^340^^,^^341^) deserve careful consideration, as they profoundly affect key salt properties. For example, Li et al.^335^ identified two polymorphs of the lamotrigine-tolfenamic acid salt (Form I and Form II), with Form I exhibiting higher apparent solubility for tolfenamic acid in a pH 6.8 buffer. Similarly, salts may undergo phase transitions, including polymorphic transitions^342^^,^^343^ and transformation between anhydrate and hydrates/solvates^338^^,^^344^, etc. Among these, salt disproportionation, in which an ionized salt reverts to its neutral form, poses a significant challenge due to the consequent loss of solubility advantages. The factors influencing salt disproportionation include: (1) salt properties (*e.g.*, solubility, pH_max_), (2) formulation variables (*e.g.*, dosage forms, excipients), and (3) manufacturing conditions (*e.g.*, temperature, relative humidity, mechanical stress)^345^, ^346^, ^347^, ^348^. Weldeab et al. reported the impact of hydrated excipients using trisodium phosphate dodecahydrate (TSPD) on tartrate salt disproportionation. In closed systems at 40 or 70 °C, *in situ* moisture release from TSPD triggered significant salt disproportionation. In contrast, no disproportionation occurred in open systems where moisture could readily escape (Fig. 31)^346^. These stability risks highlight the need for practical assessment strategies in early development of salt, such as measurement of salt's saturation solubility across various pH and residue composition analysis. Complementing these experimental approaches, computational modeling and *in silico* predictions have emerged as valuable tools for forecasting salt disproportionation^349^^,^^350^. When disproportionation risk cannot be entirely avoided, the strategic incorporation of additives (*e.g.*, polymers or small molecules) has proven effective at inhibiting the disproportionation^342^^,^^345^. Hao et al.^342^ investigated two sulfadiazine-piperazine salts (SDZ-PIP I and SDZ-PIP II)^342^ and found that SDZ-PIP II rapidly converted to pure sulfadiazine within 15 s in phosphate buffer (37 °C), resulting in the loss of its solubility advantage. However, the addition of PVP K30 significantly suppressed the disproportionation, thereby enhancing sulfadiazine's bioavailability and anti-meningitis efficacy^342^.

**Figure 32:**
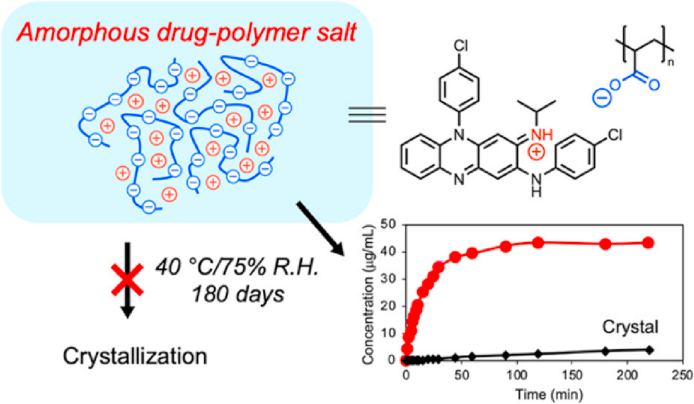
Amorphous clofazimine-poly(acrylic acid) salts with high stability under tropical conditions and fast dissolution. Reprinted with permission from Ref. 353 Copyright © 2021, The Authors.

Beyond conventional small-molecule crystalline salts, both amorphous salts and polymer salts represent promising strategies for overcoming poor aqueous solubility^337^^,^^351^^,^^352^. Kasten et al.^337^ prepared crystalline and amorphous naproxen-arginine salts and found that both salts exhibited significantly enhanced dissolution rates (14.8- and 74.1-fold, respectively) and solubility (25.3- and 29.8-fold, respectively) compared to pure crystalline naproxen. However, only the amorphous salt improved bioavailability because the crystalline salts precipitated under gastric acidic conditions. Taylor and coworkers^336^ reported that salt formation and resulting elevated *T*_g_ markedly affect the physical stability and release performance of ASDs. For instance, while lumefantrine free base formulated as a PVP/VA ASD exhibited poor physical stability, salt formation with various acids produced salt-based ASD with higher *T*_g_, improving physical stability relative to free base ASDs. However, the higher *T*_g_ of the salt-based ASDs likely required more solvent to plasticize the polymer during dissolution, resulting in limited drug release^336^. This underscores the importance of balancing *T*_g_ elevation against dissolution performance when designing amorphous salts. When the counterion itself is an ionizable polymer, the drug-polymer salts can be generated^353^, ^354^, ^355^, ^356^, ^357^, ^358^. Yu's group^356^ proposed two modes of salt formation between amorphous drugs and polymers. The first involves coating ionized drug particles with polyelectrolytes *via* an acid-base reaction^356^. Although the reaction typically forms only a thin layer on the surface of amorphous particles, it effectively inhibits crystallization by significantly reducing molecular diffusion rates at the surface. Furthermore, the polyelectrolyte coating indirectly enhances drug loading in ASDs and improves powder properties such as compressibility and flowability^357^^,^^359^^,^^360^. The second mode involves salt formation occurring within the bulk material, which confers greater physical stability and superior dissolution performance compared to neutral ASDs^353^^,^^354^^,^^356^. These improvements are attributed to the reduced molecular mobility of drugs resulting from the strong ionic interactions with polymers^361^. Gui et al.^353^ prepared an amorphous clofazimine (CFZ)-poly(acrylic acid) salt at 75% (*w*/*w*) drug loading using a slurry method. This amorphous CFZ-PAA salt demonstrated markedly greater physical stability at 40 °C and 75% RH than ASDs containing unionized CFZ dispersed in poly(vinylpyrrolidone)^353^. Salt formation with poly(acrylic acid) not only stabilized amorphous CFZ against crystallization but also ensured rapid dissolution and high solution concentrations in simulated gastric fluid and intestinal fluid (Fig. 32)^353^. Moreover, the protonated fraction of an API plays a critical role in these systems^355^. A study by Neusaenger et al.^355^ demonstrated that products prepared by slurry method, exhibiting a higher degree of salt formation, showed greater stability and release than those prepared by melt quenching with a lower degree. This study highlights that preparation methods and process conditions represent a key consideration in developing amorphous drug-polymer salts, which significantly impact product performance.

Salt formation is a practical strategy to improve the aqueous solubility of poorly water-soluble drugs. Nevertheless, its application is inherently limited to ionizable compounds, restricting its broader utility across diverse chemical entities. Counterion selection plays a pivotal role in both salt formation and downstream performance; however, beyond safety and proton transfer considerations, clear guidelines for selecting counterions to achieve specific physicochemical properties remain underexplored. Moreover, many pharmaceutical salts display high hygroscopicity and pH-dependent solubility, which can lead to deliquescence or disproportionation, compromising formulation stability. Establishing structure–property relationships between counterions and salt properties will facilitate the rational design of salts with tailored attributes. Investigating disproportionation mechanisms under physiological conditions will also guide formulation strategies to maintain solubility advantages *in vivo*. Meanwhile, greater emphasis on salt permeability, which is closely linked to solubility, could further optimize the biopharmaceutical performance of poorly water-soluble drugs^362^. Lastly, complex salt systems, such as amorphous salts and polymer salts, hold significant potential for simultaneous enhancements of dissolution rate and stability.

## 11 Concluding remarks and future perspectives

Crystal engineering represents a powerful and versatile strategy for molecular solid-state design, offering precise control over the physicochemical properties of poorly water-soluble drugs. By rationally manipulating crystal structures, this approach can enhance the solubility and dissolution rates, while maintaining the pharmacological activity of drugs. These improvements ultimately lead to superior bioavailability and therapeutic effect, positioning crystal engineering as an essential tool in modern drug development.

Despite its potential, significant challenges remain in translating crystal engineering principles into practical pharmaceutical applications. A fundamental requirement is a deep understanding of structure‒property relationships, which underpins the rational design of solid-state forms with tailored physicochemical characteristics and pharmaceutical performance. Additionally, machine learning has become increasingly pivotal in crystal engineering, enabling the prediction of polymorphs, cocrystal coformers, and amorphous formulations. As a result, AI-driven computational approaches are now essential in advancing crystal engineering strategies in the development of poorly water-soluble drugs. But there are still some problems and challenges that need to be addressed for improving the model accuracy^363^. A fundamental limitation is the scarcity of high-quality datasets for training and validating AI models. Bridging this data gap is critical for ensuring reliable AI implementation. Automated workstations with high-throughput crystallization screening enable efficient high-quality data collection by generating highly accurate and reproducible experimental results through the elimination of operator errors. Additionally, to fully realize AI's potential, integrating physics-informed simulations with experimental data offers a promising solution. This approach not only enhances model interpretability but also optimizes learning processes, reduces anomalous outputs, and improves prediction reliability. Another challenge lies in the complexity of solid form formation, which involves numerous interdependent parameters that vary across systems, scales, and operating conditions. Developing universal models capable of capturing such complexity remains an ongoing hurdle. Moreover, in solid dosage formulations, excipient selection plays a critical role in determining key product attributes such as stability, dissolution behavior, and permeability. While traditional excipient screening relies on time-consuming trial-and-error methods, a paradigm shift toward science-driven pharmaceutical formulation is needed. This requires mechanistic investigations into how excipients influence product properties, coupled with the accelerated development of computational and AI-powered predictive tools.

Another critical challenge lies in establishing robust correlations between *in vitro* dissolution/release and *in vivo* absorption. To address this, physiologically based pharmacokinetic (PBPK) modeling platforms, such as Simcyp and GastroPlus, integrate species-specific physiological and anatomical parameters with API physicochemical properties and formulation characteristics. These platforms provide comprehensive predictions of pharmacokinetic parameters, including absorption, distribution, metabolism, and elimination (ADME) processes, across virtual population cohorts, thereby bridging the gap between solid-form development and clinical performance^364^.

The preparation process and quality control of various solid forms during the manufacturing are crucial as they directly impact drug scalability, stability, bioavailability and commercial production feasibility. In recent years, continuous crystallization has emerged as a transformative technology in pharmaceutical manufacturing, particularly propelled by the FDA's strong advocacy for continuous manufacturing. Compared with traditional batch processes, continuous crystallization offers significant advantages, including shorter production cycles, higher economic benefit, and consistent product quality through PAT^365^. Recent advances have demonstrated the feasibility of continuous cocrystal manufacturing by integrating computational modeling and process analytics^115^. Future studies should prioritize advancing computational intelligence-driven data processing and novel high-sensitivity real-time monitoring technologies to enhance PAT efficiency in complex dosage manufacturing.

In conclusion, this review comprehensively examines crystal engineering strategies, covering their fundamental principles, intermolecular interaction mechanisms, classification frameworks, comparative advantages and emerging predictive methodologies. By integrating these technologies with a deeper mechanistic understanding of crystal design, the field can accelerate the translation of theoretical strategies into practical pharmaceutical applications.

## Author contributions

An Chen: Writing–review & editing, Writing–original draft. Yayun Peng: Writing–review & editing, Writing–original draft. Zhuangzhuang Chen: Writing–review & editing, Writing–original draft. Yi Lu: Writing–review & editing, Writing–original draft. Minshan Guo: Writing–review & editing, Writing–original draft, Validation, Supervision, Formal analysis, Conceptualization. Ting Cai: Writing–review & editing, Writing–original draft, Validation, Supervision, Funding acquisition, Formal analysis, Conceptualization.

## Declaration of generative AI and AI-assisted technologies in the writing process

During the preparation of this work, the authors used DeepSeek in order to improve the readability and language of the article. After using this tool, the authors reviewed and edited the content as needed and take full responsibility for the content of the publication.

## Conflicts of interest

The authors declare no conflicts of interest.
